# Protein Metabolism Underlying Heat Tolerance in Contrasting Creeping Bentgrass Lines: Insights From Gel‐Free Proteomics and Polyubiquitin‐Omics

**DOI:** 10.1111/ppl.70568

**Published:** 2025-10-10

**Authors:** Qianqian Fan, Chau‐Wen Chou, David Jespersen

**Affiliations:** ^1^ Department of Crop and Soil Sciences University of Georgia Griffin Georgia USA; ^2^ Proteomics and Mass Spectrometry Facility University of Georgia Athens Georgia USA; ^3^ Institute of Plant Breeding, Genetics and Genomics University of Georgia Griffin Georgia USA

**Keywords:** creeping bentgrass, gel‐free proteomics, heat stress, polyubiquitin‐omics, proteostasis, the ubiquitin–proteasome system, turfgrass

## Abstract

One of the major disfunctions in heat‐stressed plants is enhanced protein damage. Creeping bentgrass (*Agrositis stolonifera* L.) is an economically important perennial grass species but it is sensitive to high temperatures. Several experimental lines of creeping bentgrass varied in response for physiological traits, total protein content, and protein degradation rates in response to heat stress. The ubiquitin‐proteasome system plays a crucial role in the removal of damaged proteins, and there is a critical need to better understand the changes in proteins that occur during heat stress. Hence, we aimed to estimate change in global protein accumulations by performing gel‐free proteomics, and polyubiquitin‐omics to identify proteins that have been polyubiquitinated and targeted to the ubiquitin‐proteasome system with Tandem Ubiquitin Binding Entities in contrasting lines exposed to heat. We found that metabolic processes, like photosynthesis, antioxidant defense and protein refolding could be regulating heat tolerance in creeping bentgrass. Heat‐tolerant line S11 729‐10 was able to maintain proteins involved in the light reactions of photosynthesis, while enhancing antioxidant proteins, particularly during the later phase of heat stress. This contributed to its improved performance, including greater cell membrane integrity as well as healthier light harvesting components. Additionally, the faster turnover of key polyubiquitinated antioxidant proteins in S11 729‐10 likely represents a critical mechanism for protecting against oxidative damage. This is the first time that polyubiquitin‐omics has been utilized in turfgrass. These findings provide insights into protein metabolism during heat stress that could be utilized to help develop new cultivars with enhanced tolerance to heat.

## 1 Introduction

Maintenance of protein metabolism is of utmost importance for the normal cellular activities of plants as proteins are the major driving force behind plant growth, development, and stress tolerance. Unfavorable environmental conditions, however, can induce proteome disruption. One such stressor is heat stress, which is a major threat to many economically important plant species, with damages being further exacerbated with more frequent and intense heat wave events as a function of climate change (Pachauri and Meyer 2014). Heat stress leads to large‐scale protein misfolding and aggregation, resulting in a decline in protein content, with concomitant impairment of various physiological activities, including inhibition of photosynthesis caused by the degradation of the photosynthetic machinery, plus oxidative stress resulting from the overproduction of reactive oxygen species (ROS; Goldberg 2003; Fan and Jespersen 2023). To prevent cellular dysfunction and injuries to cells, plants can adjust their proteome to inhibit the formation or promote the removal of these damaged proteins. These mechanisms include refolding by heat shock proteins (HSPs) or degradation through various proteolytic pathways, including the ubiquitin‐proteasome system (UPS). HSPs are well‐known molecular chaperones that work by preventing proteins from inappropriate aggregation and promoting refolding of damaged proteins to maintain their functional conformations (Parsell and Lindquist 1993). Many HSPs are initially induced upon temperature elevation, contributing to protein stabilization under stressful conditions (Huang et al. 2014). Similar to HSPs, a series of antioxidant enzymes and nonenzymatic antioxidants are also upregulated in response to heat stress to scavenge excessive ROS, protecting cellular components like proteins from being oxidized and maintaining cell membrane stability (Huang et al. 2014). Despite the observed accumulation of heat‐protective proteins, other essential proteins, such as those involved in photosynthesis and electron transport, are often downregulated by heat, suggesting impairment to light‐dependent reactions (Huang et al. 2014; Wang, Xu, et al. 2017; Zhao and Hou 2023). These differential changes in the proteome demonstrate that plants' response to environmental perturbation is an intricate process at the molecular level. A better understanding of the changes in protein accumulation will assist in dissecting physiological responses, thereby providing deeper insights into the molecular basis of heat tolerance.

Proteomics has been a powerful approach to discover the proteins and pathways that are crucial for stress tolerance and has been addressed in a wide range of plant species, including creeping bentgarss (*Agrositis stolonifera* L.; Huang et al. 2014; Lyon et al. 2016; Ali et al. 2023; Zhao and Hou 2023; Sinha et al. 2024). Creeping bentgrass, an economically important perennial grass species, is largely used on high‐value turf areas such as putting greens on golf courses and courts for tennis lawns given its fine texture and tolerance to low mowing height (Fry and Huang 2004). However, due to its low to moderate tolerance to high temperatures, heat stress has been one major factor limiting the performance of creeping bentgrass worldwide (Fry and Huang 2004). To further understand thermotolerance mechanisms and accelerate the development of elite cultivars with enhanced tolerance levels, attempts have been made to investigate proteomics in heat‐stressed creeping bentgrass (Huang and Xu 2008; Xu and Huang 2010; Jespersen and Huang 2015; Li, Zeng, et al. 2020). It was found from these studies that compared to heat‐sensitive lines/cultivars, heat‐tolerant ones had lesser extents of downregulation of the proteins involved in important pathways like photosynthesis and energy metabolism, accompanied by greater upregulation or even unique induction of stress‐responsive proteins like HSPs and certain antioxidants. These collectively contributed to the enhanced thermotolerance in the heat‐tolerant plants. Nevertheless, the majority of these studies use older gel‐based methods prior to the development of more advanced mass‐spectrometry‐based tools. Gel‐free proteomics, as a relatively newer methodology, possesses several advantages over gel‐based ones, such as a broader dynamic range and greater sensitivity, higher reproducibility, improved quantification accuracy, and more efficient protein identification (Zargar et al. 2016). Exploring protein metabolism from the point of gel‐free proteomics, thus, may offer some new insights into the survival strategies creeping bentgrass utilizes to cope with heat stress.

The UPS is a key proteolytic pathway responsible for maintaining protein homeostasis by targeting damaged proteins for degradation, primarily in the cytosol and nucleus, but also in organelles like chloroplasts and the endoplasmic reticulum (Wu and Rapoport 2018; Sun et al. 2022). It relies on a cascade of ubiquitin enzymes—E1, E2, and E3—to attach ubiquitin molecules to substrate proteins, forming polyubiquitin chains that are recognized and degraded by the 26S proteasome (Xu et al. 2024). This process eventually results in the release of free amino acids and ubiquitin molecules to be reused for various cellular functions. Given the importance of the UPS in protein quality control, there has been a growing body of research that investigated its role in plant responses to various abiotic stresses, including heat stress (Shang and Taylor 2011; Zhang, Li, et al. 2015; Doroodian and Hua 2021; Xu et al. 2024; Fan and Jespersen 2025a, 2025b). In the case of tobacco ( *Nicotiana tabacum* L.) cells, heat‐induced increases in UPS activity have been observed (Paradiso et al. 2020). Similarly, another study on wheat ( *Triticum aestivum* ) proposed the accelerated breakdown of root proteins via an enhanced UPS pathway, as manifested by elevated amounts of ubiquitin‐protein conjugates and lower amounts of free ubiquitin found in cells (Ferguson et al. 1990). The integration of proteomics with protein ubiquitylation, referred to as ubiquitin‐omics, is a relatively new and promising area of research. It enables the large‐scale identification of substrate proteins targeted by the UPS, offering valuable insights into the regulation of key stress‐responsive proteins and various biological processes (BPs), potentially contributing to enhanced proteostasis, thereby increasing stress tolerance in plants (Xu et al. 2024). Despite its significance, identifying UPS substrates has been challenging due to the transient nature of ubiquitination and the dynamic interactions between substrates and E3 ligases. However, ubiquitin‐omics has emerged as a powerful tool to overcome these challenges, which has been applied to a variety of plant species, such as maize ( *Zea mays* ), *Arabidopsis thaliana* and rice ( *Oryza sativa* L.; Fan et al. 2021; Berger et al. 2022; Ying et al. 2023). The key step of this procedure is the efficient enrichment of ubiquitin conjugates. Traditional strategies for the isolation of polyubiquitinated proteins often require immunoprecipitation of epitope‐tagged ubiquitin, which, however, displays a lack of affinity for polyubiquitin chains (Olzmann and Chin 2008). Tandem Ubiquitin Binding Entities (TUBEs), which are an engineered protein domain, can overcome this problem. This technology shows greater affinity for polyubiquitin chains than most ubiquitin antibodies and is emerging as an indispensable strategy for further understanding of the UPS (Kadimisetty et al. 2021). Integrating TUBE‐based isolation of polyubiquitinated proteins with proteomics, thus, may offer new insights into how protein degradation is regulated by the UPS under heat stress.

Recent studies on several promising experimental lines of creeping bentgrass revealed various tolerance levels to heat stress, with differential responses identified in terms of physiological traits and total protein contents (Fan and Jespersen 2023). Stronger activities of the proteolytic machinery, including the UPS, were generally detected in response to temperature elevation (Paradiso et al. 2020). However, the substrate proteins for the UPS pathway and how these individual proteins were regulated under heat stress remain elusive. The development of elite cultivars will be more efficient with a more complete understanding of the mechanisms conferring improved thermotolerance. There is a critical need to better understand the changes in protein accumulation that are driving the differences in heat tolerance as well as the underlying regulation of these changes. Hence, we aimed to estimate global proteomic changes due to heat stress by performing gel‐free proteomics in contrasting creeping bentgrass lines and identify proteins that have been polyubiquitinated and targeted to the UPS pathway via polyubiquitin‐omics. Findings from this study would enable a more complete picture of protein metabolism underpinning thermotolerance and identifying stress‐related proteins and pathways that can be utilized for new cultivar development.

## 2 Materials and Methods

### 2.1 Growth and Treatment Conditions

Three creeping bentgrass lines with varying levels of heat stress tolerance were selected for this study based on previous findings (Fan and Jespersen 2023). These included two experimental lines (“S11 675‐02” and “S11 729‐10”) and one commercial cultivar (“Crenshaw”), with S11 729‐10 being heat‐tolerant while S11 675‐02 and Crenshaw being heat‐sensitive. For each line, 6‐cm‐diameter plugs were established in plastic pots (10.5 cm long, 10.5 cm wide, and 12.5 cm deep) filled with a mixture of 50% sand and 50% calcined clay (Turface; Profile Products LLC), and placed in greenhouse conditions with ~23°C/~15°C (light/dark period temperatures) and 70% relative humidity for 2 months. Then the plants were transferred to controlled environmental growth chambers (CG‐72; Conviron) for 1‐week acclimation under conditions of 20°C/15°C (day/night), 70% humidity and 14 h photoperiod with a 600 μmol m^−2^ s^−1^ photosynthetically active radiation at the canopy level before the treatments began. Plants were maintained well‐watered and fertilized weekly with a 24‐8‐16 (N‐P‐K) fertilizer (Scotts Miracle‐Gro) at the rate of 9.8 g N m^−2^ during establishment in the greenhouse as well as during the treatment period inside the growth chambers. Applications of insecticides and fungicides were made as needed for disease control. Plants of each line were placed under either heat stress (38°C/33°C day/night) or control (20°C/15°C day/night) conditions for 28 days after the initiation of the treatments. These temperatures are widely utilized to induce heat stress in C3 perennial grass species (Jespersen et al. 2016; Wang, Juliani, et al. 2017; Du et al. 2025; Fan and Jespersen 2025b).

### 2.2 Physiological Measurements

Weekly measurements of visual turf quality (TQ) rating, percent green cover, electrolyte leakage (EL), and chlorophyll fluorescence were performed. TQ and green cover represent overall turf performance. TQ was rated on a scale of 1–9 according to color, density and uniformity with 1 representing totally dead grass, while 9 standing for completely healthy grass (Krans and Morris 2007). Percent green cover was acquired through images taken with a digital camera (Canon G9X; Canon) using a lightbox to ensure a uniform lighting, which were then processed via ImageJ v.1.46. (Karcher and Richardson 2013).

Chlorophyll fluorescence reflects the health status of photosynthetic light harvesting. To conduct chlorophyll fluorescence measurements, plants were dark‐adapted for at least 30 min prior to measurement via a chlorophyll fluorometer (OSP 5+; Opti‐sciences). Fluorescence traits consisted of absorbed energy flux per cross section (ABS/CSm), quantum efficiency of energy dissipation in photosystem II (PSII) antenna (DIo/ABS), quantum efficiency of energy flux trapped by PSII photochemistry leading to reduction of quinone A (Q_A_) (Fv/Fm), the energy flux associated with electron transport from Q_A_ to intersystem electron acceptors such as plastoquinone pool per cross section (ETo/CSm), and the energy flux associated with electron transport from intersystem electron acceptors to final PSI acceptors per cross section (REo/CSm; Stirbet et al. 2018). Three measurements were taken on fully expanded leaves for each replicate.

Cell membrane stability, as estimated by EL, is widely used as an indicator for membrane damage in plants (Fan and Jespersen 2023). To quantify EL, around 0.1 g fresh leaves were placed in a tube containing 35 mL deionized water. After agitating tubes on a shaker for 16 h, initial conductivity was recorded through a conductivity meter (Radiometer). Next, the samples were autoclaved at 120°C for 20 min, followed by incubation for another 16 h on a shaker, after which the final conductivity was read. EL then was calculated as the percentage of initial conductivity over total conductivity (Blum and Ebercon 1981).

### 2.3 Polyubiquitin‐Omics and Gel‐Free Proteomics

Gel‐free proteomics for global proteins at 14 and 28 days and polyubiquitin‐omics at 28 days were performed by the Proteomics and Mass Spectrometry Facility at the University of Georgia. Since the most significant differences in physiological responses were found between Crenshaw and S11 729‐10, both proteomic analyses focused on these two lines for comparison. Approximately 500 mg leaf tissues were harvested from each plot at 14 and 28 days and immediately flash‐frozen in liquid nitrogen. Frozen leaves were stored at −80°C until further analysis.

#### 2.3.1 Global Protein Extraction, Purification, and Digestion

Proteins were extracted according to the trichloroacetic acid/acetone method with minor modifications (Niu et al. 2018). Around 150 mg leaves of each sample were homogenized in 1.5 mL sodium phosphate buffer (50 mM, pH 7.5 with 1 mM ethylenediaminetetraacetic acid), followed by centrifugation (10,000*g*, 4°C for 20 min) to obtain 0.4 mL supernatant containing crude proteins. The crude protein supernatant was transferred to a solution containing 20% TCA and 0.14% 2‐mercaptoethanol in acetone (pH 7.5). The mixture was fully vortexed, placed on ice for 5 min, and then centrifuged (15,000*g* for 3 min). The resultant tissue pellets were washed using 0.07% 2‐mercaptoethanol in acetone and 0.07% 2‐mercaptoethanol in 80% acetone, successively, then air dried for 9 min at 37°C. The final pellets were resuspended by vortexing at 25°C in 0.4 mL resolubilization buffer (7 M urea, 2 M thiourea, 5% (m/v) CHAPS, and 2 mM tributylphosphine, pH 7.5), followed by centrifuging at 21,000*g*, 25°C for 20 min. The resulting supernatant was collected as protein extracts. Total protein content was quantified at 595 nm using a Bradford dye reagent with a bovine serum albumin standard (Bradford 1976).

Proteins were further purified according to a modified filter‐assisted sample preparation (Nagaraj et al. 2008). Ten microgram of protein sample was mixed with 5 μL of 0.05 M dithiothreitol and then denatured by a heat block at 100°C for ~10 min. After cooling down to room temperature, the mixture was incubated for an additional 30 min. The protein samples were diluted with 100 μL of 20 mM triethylamine bicarbonate (Millipore‐Sigma) and then transferred into Vivacon 500 (10 K MWCO Hydrosart) filters (Sartorius). The filter units were spun at 14,000*g* in a microcentrifuge (Sorvall Legend Micro17, Thermo Scientific) for 15 min, and the flow‐throughs were discarded. The resultant proteins were alkylated by adding 50 μL of 0.02 M iodoacetamide to the filters and were allowed to react in the dark for 30 min. The filters were washed/spun with 400 μL of 20 mM triethylamine bicarbonate for two times. Then 0.2 μg of Trypsin in 50 μL of 20 mM triethylamine bicarbonate was dispensed into each filter to digest the proteins at room temperature overnight. The next day, the filters were spun at 14,000*g* for 10 min to allow for the elution of tryptic peptides to the collection tubes, followed by a wash with 100 μL of water to elute the residual peptides. The collected peptide solutions were dried in a vacuum concentrator (RC1010 centrifuge, Jouan).

#### 2.3.2 Enrichment, Purification and Digestion of Polyubiquitinated Proteins

Polyubiquitinated proteins were isolated according to TUBE technology using the K48 version of the ubiquitin mass spectrometry kit (LifeSensors), with minor modification. Around 100 mg of leaves from each sample were homogenized in 1.0 mL of sodium phosphate buffer (50 mM, pH 7.5) with 1 mM ethylenediaminetetraacetic acid, followed by the addition of 10 μL of UPS inhibitor cocktail. After centrifuging (14,000*g*, 4°C for 20 min), 5 μL of decomplexing buffer was added to the resultant 500 μL supernatant, followed by a 15‐min incubation over ice. The total protein content was quantified at 595 nm using a Bradford dye reagent with a bovine serum albumin standard (Bradford 1976). A total of 100 μL of slurry containing magnetic beads bound with TUBEs was added to the supernatant containing 1–3 mg of total proteins, and PBS buffer was used to adjust the final volume to 1 mL. After incubation overnight at 4°C using an end‐to‐end rotator (Benchmark, Edison), the beads of the mixture were collected using a magnetic stand (VWR). Following three washes with PBS‐T and another wash with 80 μL of TUBE wash buffer, the beads were resuspended and incubated in 30 μL of TUBE elution buffer at room temperature for 15 min with mixing. The resulting eluate contained polyubiquitinated proteins.

A total of 3.3 μL neutralization buffer was added into the eluted proteins, followed by centrifugation (14,000*g*, 4°C for 5 min). Then, 4 μL of 6X Laemmli buffer (Thermo Fisher Scientific) was added into 20 μL of the resultant supernatant. After boiling it at 90°C for 5 min, the mixture was loaded into a Bolt 4%–12% acrylamide gel (Invitrogen) for SDS‐PAGE via a mini gel tank (Invitrogen) in order to remove detergents. The gel was run at 150 V for 2 min and then stained using SimpleBlue SafeStain (Invitrogen). After destaining in DI water for 1 h, the gel bands with stain were carefully excised. Gel digestion was performed in the dark. Specifically, the gel bands were sliced into small pieces, followed by rinsing with 50% acetonitrile/20 mM triethyl ammonium bicarbonate (~pH 7.5–8) twice. The gel pieces were then dehydrated by adding 100% acetonitrile and dried out by a vacuum concentrator (RC1010 centrifuge, Jouan). Next, a variable amount of Trypsin solution (0.01 μg μL^−1^ in 20 mM triethyl ammonium bicarbonate) was added until the gel pieces totally absorbed the Trypsin solution, followed by overnight incubation at 37°C. The tryptic peptides were extracted from the gel pieces by incubating with 50% acetonitrile/0.1% formic acid twice and were dried in a vacuum concentrator (RC1010 centrifuge, Jouan).

#### 2.3.3 Analysis Through Tandem Mass Spectrometry Coupled With Liquid Chromatography (LC–MS/MS)

The downstream analysis workflow for digested global proteins and polyubiquitinated proteins is performed the same for both types of samples as described in Sections 2.3.3 and 2.3.4. The mass spectrometry analyses were performed on a Thermo‐Fisher LTQ Orbitrap Elite Mass Spectrometer coupled with a Proxeon Easy NanoLC system (Waltham). Approximately 0.5 μg peptides were loaded into a reversed‐phase column (100 μm inner‐diameter, approximately 15 cm long, ReperoSil‐Pur 120 C18‐AQ, Dr. Maisch) and then directly eluted into the mass spectrometer for a 90‐min run. A two‐buffer gradient elution consisting of 0.1% formic acid as buffer A and 99.9% acetonitrile with 0.1% formic acid as buffer B was used for analysis. The gradients were as follows: 0–2 min, 5% buffer B; 2–60 min, 20% buffer B; 60–85 min, 50% buffer B; 85–95 min, 95% buffer B. Data were acquired using Xcalibur software (version 3.0, Thermo Fisher Scientific). The data‐dependent acquisition method was used to acquire MS data. A survey MS scan was acquired first, and then the Top 10 ions in the MS scan were selected for following collision‐induced dissociation analysis. Both MS and MS/MS scans were acquired by Orbitrap at the resolutions of 120,000 and 15,000, respectively.

#### 2.3.4 Protein Identification, Quantification, and Classification

Protein identification was performed using Proteome Discoverer 3.0 (Thermo) with Mascot 2.8 (Matrix Science) against the Poaceae database of UniProt (downloaded on 12/2023) and a modified contaminations database containing commonly known contaminating proteins (Mascot). The search parameters were as follows: precursor mass tolerance 10 ppm, fragment mass tolerance 0.02 Da, trypsin full specificity, maximum number of missed cleavages 1, false discovery rate < 0.01; methionine oxidation was set as variable modifications, and cysteine carbamidomethylation was designated as fixed modification. The semiquantitative analyses were achieved using a label‐free quantification workflow within Proteome Discoverer. Specifically, a Precursor Ion Quantifier node calculated the summated peak areas of the peptide matches of protein matches in the Extracted Ion Chromatograms (mass precision, 5 ppm). Protein was identified to be differentially expressed proteins (DEPs) when it showed a fold change (FC) of no less than 1.2 or no larger than 0.83 in the heat‐stressed condition compared with the control condition with a *p*‐value ≤ 0.05 (Li et al. 2019; Li, Nadeem, et al. 2020; Li, Zeng, et al. 2020).

Gene ontology (GO) is an international classification system that describes biological functions at various levels, from molecular to organismal. The Kyoto Encyclopedia of Genes and Genomes (KEGG) is a database of manually drawn pathway maps representing our knowledge of molecular interaction and reaction networks. In our study, GO analysis including BP and molecular function (MF) was performed using the Blast2GO program (OmicsBox 3.1.11) against the nonredundant protein database (Conesa et al. 2005). KEGG pathway annotation was conducted via g:Profiler (https://biit.cs.ut.ee/gprofiler/gost, accessed on 03/2024; Kolberg et al. 2020). The pathways were identified to be enriched with a *p*‐value ≤ 0.05 using the Benjamini–Hochberg approach.

### 2.4 Statistical Analysis

A completely randomized design was adopted within each temperature. There were four replicates for each line under control and heat stress conditions, respectively. Within each temperature, two‐way ANOVA was performed by fitting a linear regression model for physiological measurements in RStudio (R 4.3.3, 2024), with both line and date as fixed effects. Before ANOVA, the normal distribution of residuals and the homogeneity of variance were checked using the gvlma package in RStudio to make sure the data met the ANOVA assumptions. Means were separated by Fisher's protected least significant difference at the 0.05 probability level. For the visualization of proteomics data, a Venn diagram, a PCA (principal component analysis) plot as well as a radar plot were made using ggvenn, ggfortify, and ggradar packages, respectively, in RStudio. Additional figures were created using either Hiplot (https://hiplot.com.cn/) or SRplot platforms (https://www.bioinformatics.com.cn/srplot).

## 3 Results

### 3.1 Overall Performance, Electrolyte Leakage, and Chlorophyll Fluorescence Traits

Temperature effects were mostly significant according to the preliminary analysis. The three‐way interaction, however, was difficult to interpret. Investigating the interaction between date and line under heat stress conditions may offer more valuable insights into tolerance differences among lines than focusing on the interaction between temperature and other treatments, due to the temporal differences in the onset of heat stress symptoms as the experiment progressed (Fan and Jespersen 2023).

**FIGURE 1:**
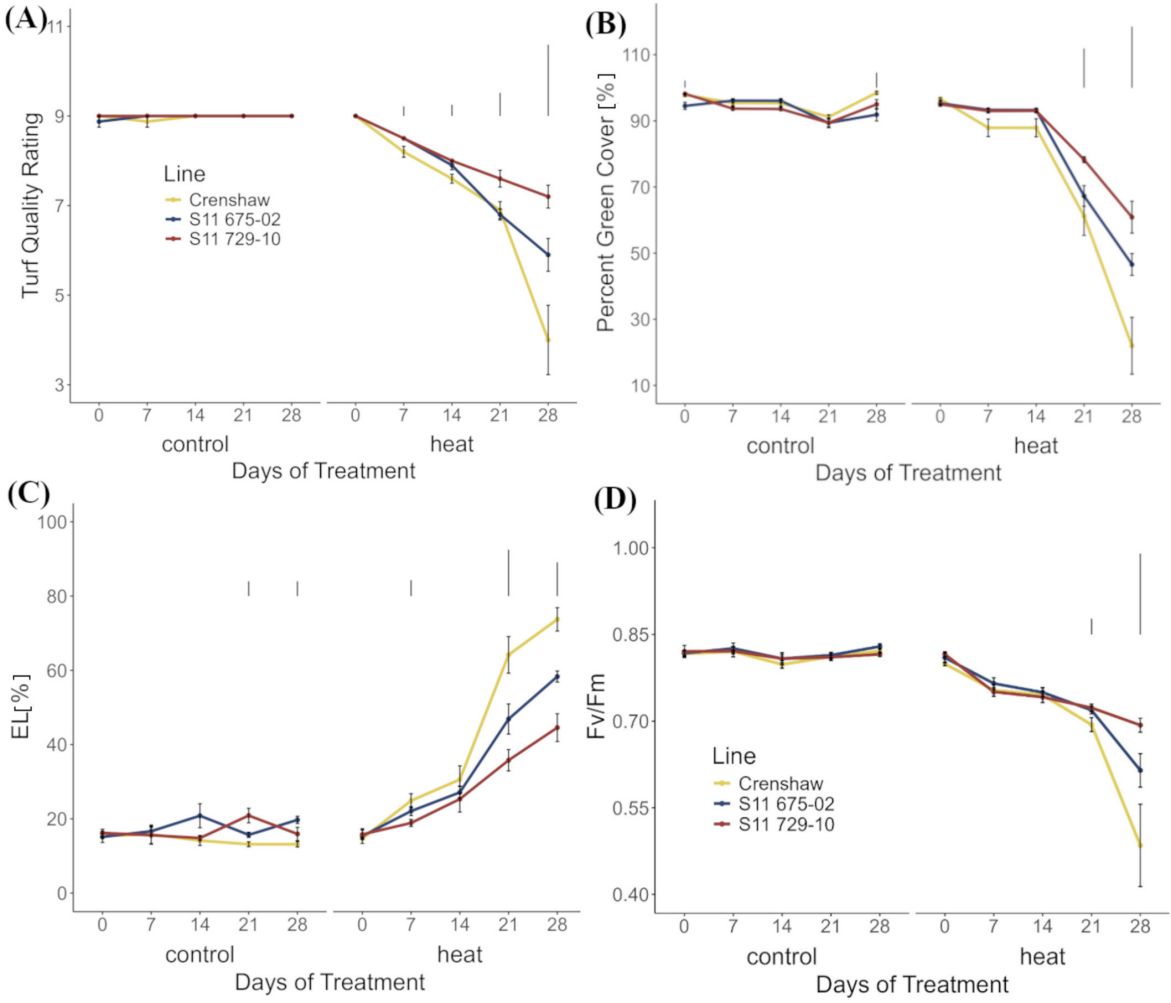
Physiological responses of creeping bentgrass over time. (A) Change in turf quality ratings, (B) green cover, (C) electrolyte leakage (EL) and (D) Fv/Fm for creeping bentgrass lines over time under control (20°C/15°C day/night) and heat stress (38°C/33°C day/night) conditions. Data are presented as means ± standard errors. Bars above lines represent LSD values at *p* = 0.0 determined on days when significant differences among lines were found. Fv/Fm, quantum efficiency of energy flux trapped by Photosystem II photochemistry.

In contrast to control conditions, heat stress caused dramatic decreases in TQ and green cover (up to 55.6% and 77.2% declines, respectively, relative to 0 days) while significant increases in EL (up to 401.4% relative to 0 days) occurred over time (Figure 1A–C; Table S1). Prominent separations among lines were observed from the second measurement at 7 days for TQ and EL, and from the fourth measurement at 21 days for green cover. At the end of the trial (Figure S1), Crenshaw was the worst performer with the lowest TQ and green cover, while having the highest EL. No significant differences were found between the other two lines in terms of TQ and green cover. However, S11 729‐10 showed significantly lower values of EL than S11 675‐02 at 28 days.

**FIGURE 2:**
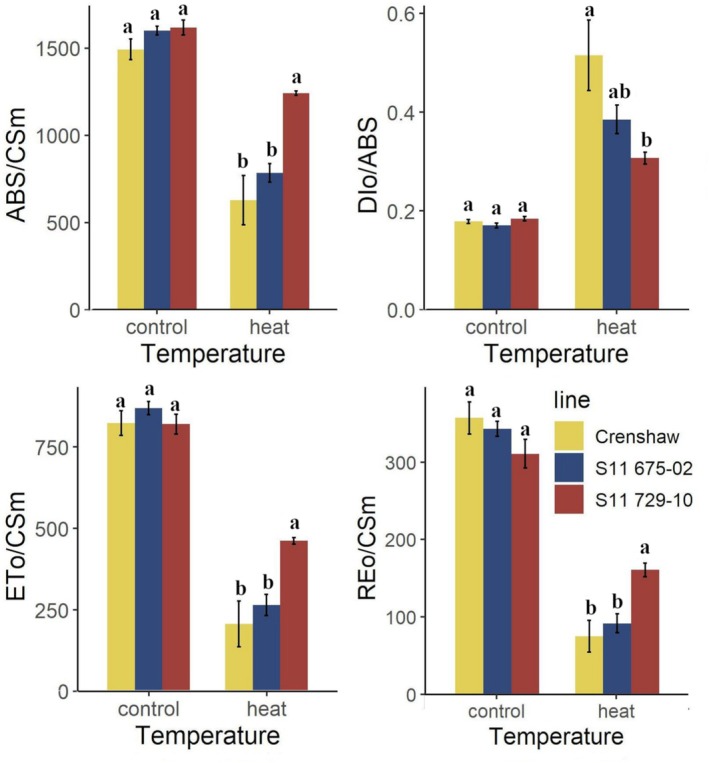
Chlorophyll fluorescence traits for creeping bentgrass lines under control (20°C/15°C day/night) and heat stress (38°C/33°C day/night) conditions at 28 days. Data are presented as means ± standard errors. Columns marked with the same lowercase letters are not significantly different at *p* = 0.05 within each temperature condition. ABS/CSm, absorbed energy flux per cross section; DIo/ABS, quantum efficiency of energy dissipation in PSII antenna; ETo/CSm, the energy flux associated with electron transport from quinone A to intersystem electron acceptors such as plastoquinone pool per cross section; REo/CSm, the energy flux associated with electron transport from intersystem electron acceptors to final photosystem I acceptors per cross section.

Similar to TQ and green cover, Fv/Fm was reduced by heat stress throughout the trial with the greatest extent of decrease (up to 40.0% relative to 0 day) seen in Crenshaw (Figure 1D, Table S5). Additional differences were detected during the later phase of stress (21 and 28 days). At 28 days, S11 729‐10 was the best line whose Fv/Fm value was 43.1% higher than Crenshaw. No significant differences were seen between S11 729‐10 and S11 675‐02, or between Crenshaw and S11 675‐02 at 28 days of heat stress. In addition to Fv/Fm, other traits indicating various light harvest processes, also showed significant changes in response to heat stress, with declines observed for ABS/CSm, ETo/CSm, and REo/CSm while increases were detected for DIo/ABS in all the three lines (Figure 2). Again, S11 729‐10 outperformed others in terms of these traits at 28 days, having significantly higher values relative to Crenshaw and S11 675‐02 for ABS/CSm, ETo/CSm and REo/CSm, while showing significantly lower values relative to Crenshaw for DIo/ABS.

### 3.2 Gel‐Free Proteomics

**FIGURE 3:**
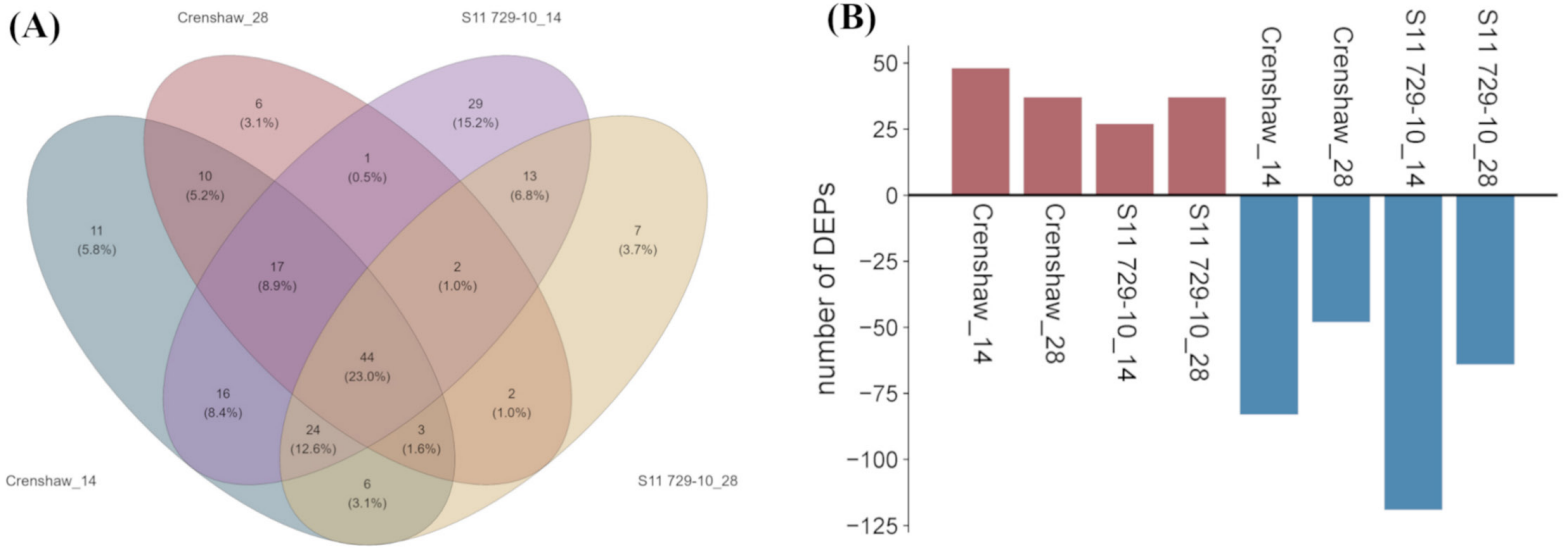
Overall change in differentially expressed proteins (DEPs) of two creeping bentgrass lines (Crenshaw and S11 729‐10) at 14 and 28 days. (A) Venn diagram showing common and unique DEPs among different groups. (B) Bar plot showing upregulated and downregulated proteins for each group. Red bars represented upregulation while blue bars represented downregulation.

Out of a total of 373 putative proteins identified, 240 proteins were successfully quantified via LC–MS/MS. Specifically, 131, 85, 146, and 101 proteins were considered to be DEPs in the heat‐stressed group compared with the control group for Crenshaw at 14 days, Crenshaw at 28 days, S11 729‐10 at 14 days, and S11 729‐10 at 28 days, respectively (Figure 3A, Tables S6–S9). Forty‐four DEPs were commonly shared across different groups, while 11, 6, 29, and 7 DEPs were uniquely found for Crenshaw at 14 days, Crenshaw at 28 days, S11 729‐10 at 14 days and S11 729‐10 at 28 days, respectively. For Crenshaw, at 14 days, 48 DEPs were upregulated while 83 were downregulated. At 28 days of heat stress, 37 DEPs were upregulated while 48 were downregulated in Crenshaw compared to the control (Figure 3B). For S11 729‐10, at 14 days of heat stress, 27 DEPs were upregulated while 119 were downregulated compared to the control; at 28 days of heat stress, 37 DEPs were upregulated in S11 729‐10 while 64 were downregulated compared to the control. Additionally, PCA showed a clear separation in terms of DEPs between heat‐stressed and control samples for each line at a given day of treatment (Figure S2).

**FIGURE 4:**
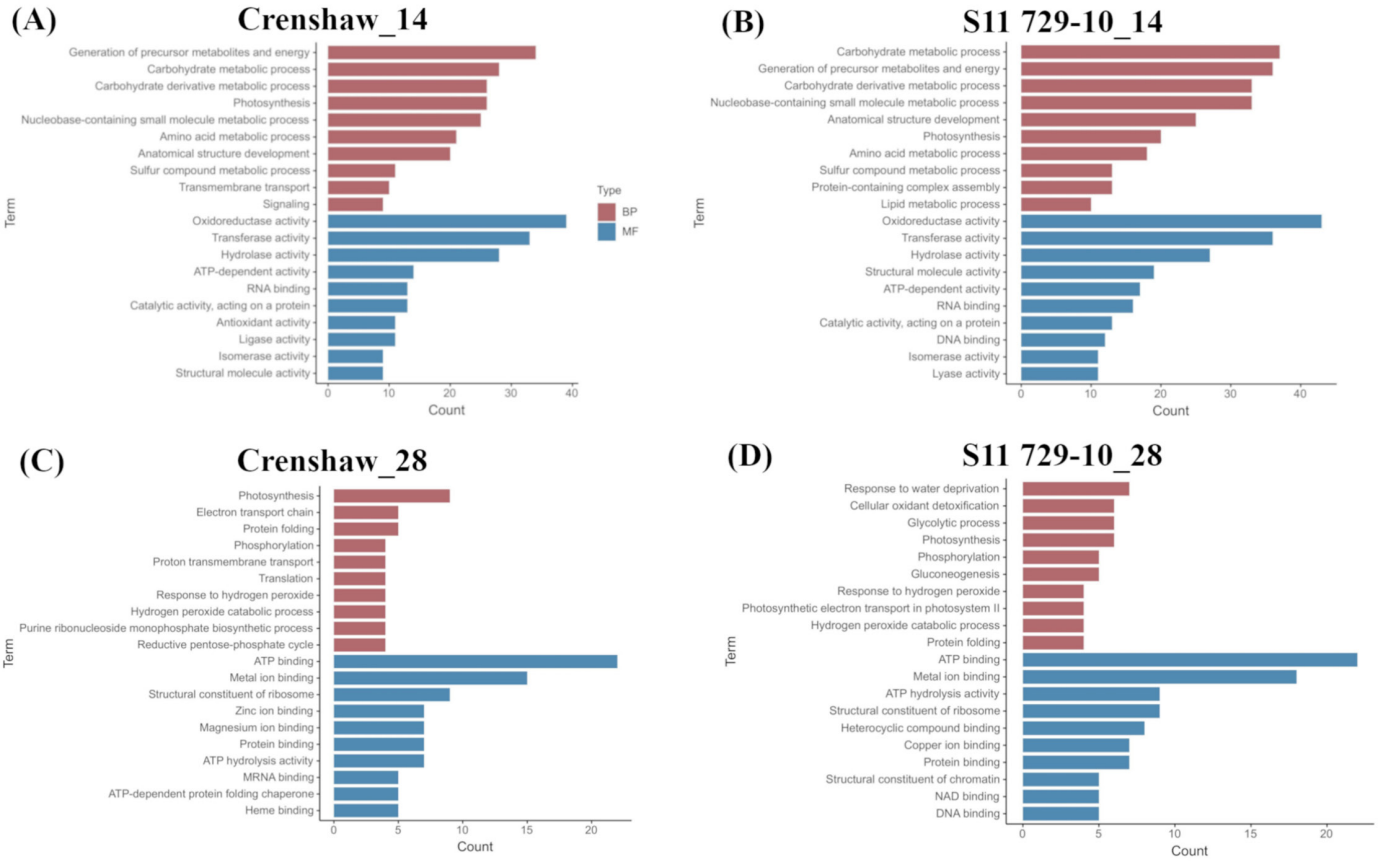
Gene ontology (GO) of heat responsive proteins. Top 20 GO terms for differentially expressed proteins (DEPs) of two creeping bentgrass lines (Crenshaw and S11 729‐10) at 14 days (A, B) and 28 days (C, D) responding to heat stress. Count was the number of DEPs falling into each GO term. BP, biological process; MF, molecular function.

To evaluate the function of DEPs, GO analysis was performed. The distribution of the Top 10 GO terms for BP and MF, respectively, mostly overlapped across lines within each time point (Figure 4). At 14 days, top BP categories included the generation of precursor metabolites and energy, carbohydrate metabolic process, carbohydrate derivative metabolite process, photosynthesis, and nucleobase‐containing small molecule metabolic process, whereas the top MF categories included oxidoreductase activity, transferase activity, hydrolase activity and ATP‐dependent activity. At 28 days, the most represented GO terms for BPes were typically photosynthesis, phosphorylation, response to hydrogen peroxidase, electron transport and protein folding across lines. Nevertheless, response to water deprivation, glycolytic process and gluconeogenesis were uniquely included in the Top 10 terms for S11 729‐10. Regarding MFs, the top GO categories associated with DEPs were ATP binding, metal ion binding, structural constituent of ribosome as well as protein binding at the end of the trial (28 days).

To investigate the involvement of DEPs in important heat‐responsive pathways, DEPs within each group were annotated according to the KEGG database using gProfiler. A total of 77, 89, 53, and 62 proteins were assigned KEGG annotations in the heat‐stressed samples for Crenshaw at 14 days, Crenshaw at 28 days, S11 729‐10 at 14 days, and S11 729‐10 at 28 days, respectively. These represented 58.8%, 62.4%, 61.0%, and 61.4% of all DEPs within their respective groups (Figure S3). These DEPs with KEGG annotation were enriched into 13, 16, 12, and 11 pathways for Crenshaw at 14 days, Crenshaw at 28 days, S11 729‐10 at 14 days, and S11 729‐10 at 28 days, respectively. Most DEPs associated with KEGG pathways were classified into carbohydrate metabolism like glyoxylate and dicarboxylate metabolism, energy metabolism including photosynthesis and carbon fixation, as well as amino acid metabolism such as arginine biosynthesis or alanine, aspartate, and glutamate metabolism. Additionally, the majority of them overlapped across different groups. To maximize the number of DEPs within annotated pathways, for those DEPs without KEGG annotation, their sequences were blasted against the UniProt database to determine putative functions and pathways. Similarly, most DEPs fell into carbohydrate metabolism, energy metabolism and amino acid metabolism after combining results from gProfiler and UniProt blast results.

**FIGURE 5:**
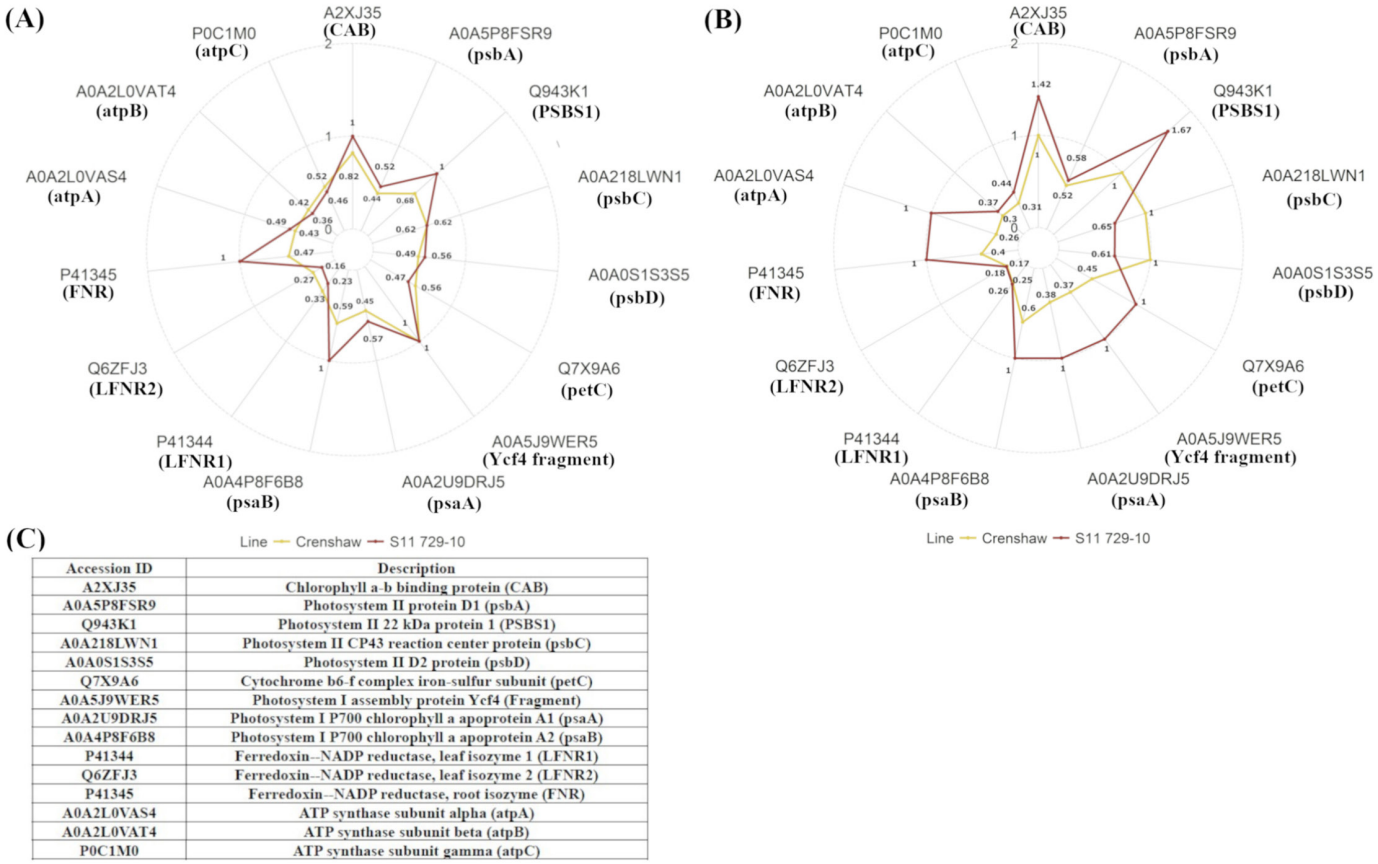
Fold change of the differentially expressed proteins involved in the photosynthesis–electron transport chain for creeping bentgrass (Crenshaw and S11 729‐10) when heat‐stressed samples were compared against control samples at 14 days (A) and 28 days (B). The corresponding protein description, including full name and short name for each accession ID, was attached (C).

In terms of photosynthesis‐light reactions, heat stress caused dramatic downregulation of all related DEPs except Photosystem I assembly protein Ycf4 (A0A5J9WER5) in Crenshaw at 14 days, with FC ranging from 0.16 to 0.82 (Figure 5A). Comparatively, in S11 729‐10 at 14 days, the number of downregulated proteins and the extent of decrease were both smaller. When heat stress progressed into Day 28, these DEPs were mostly further decreased in Crenshaw, while in S11 729‐10, some of them showed no significant change or even upregulation compared to control conditions (Figure 5B,C). These DEPs related to the light‐dependent reactions of photosynthesis included cytochrome b6‐f complex iron–sulfur subunit [Q7X9A6], Photosystem I assembly protein Ycf4 [A0A5J9WER5], Photosystem I P700 chlorophyll a apoprotein A1 [A0A2U9DRJ5], Photosystem I P700 chlorophyll a apoprotein A2 [A0A4P8F6B8], Ferredoxin–NADP reductase [P41345], ATP synthase subunit alpha [A0A2L0VAS4], Chlorophyll a–b binding protein [A2XJ35], and PSII 22 kDa protein 1 [Q943K1].

**FIGURE 6:**
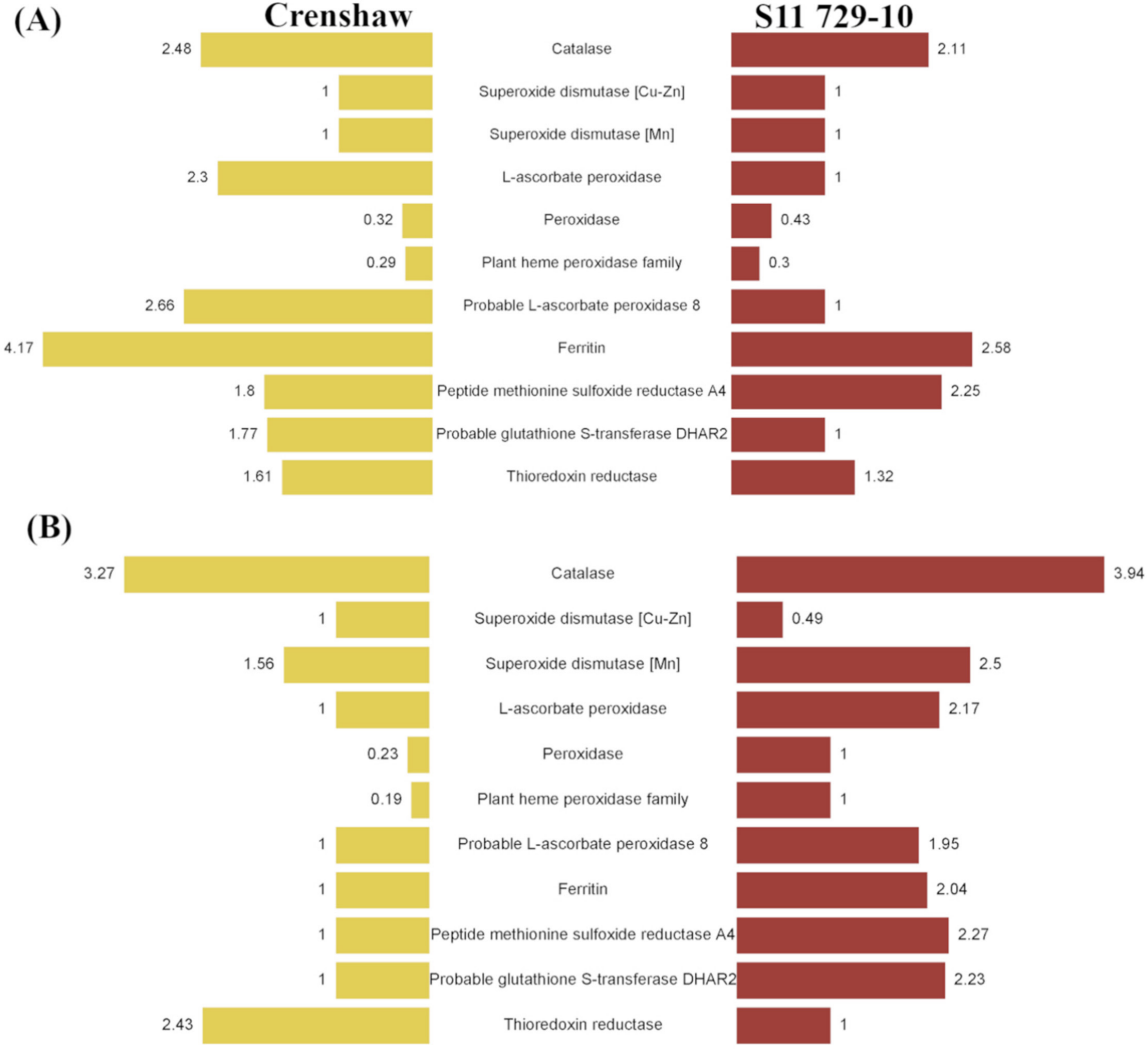
Fold change of the differentially expressed proteins involved in antioxidant defense for creeping bentgrass (Crenshaw and S11 729‐10) when heat‐stressed samples were compared against control samples at 14 days (A) and 28 days (B). Yellow represented Crenshaw while red represented S11 729‐10.

**FIGURE 7:**
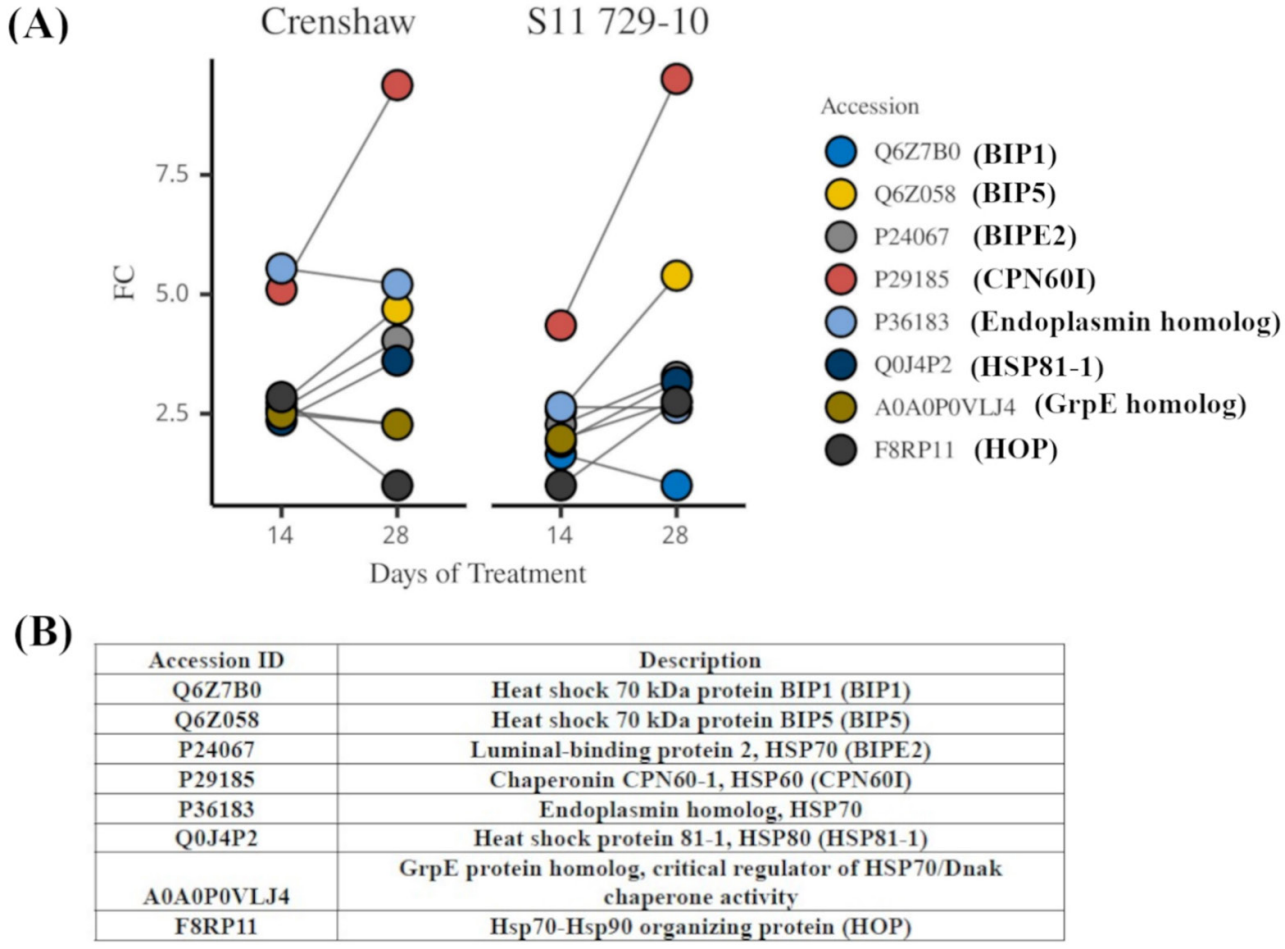
Fold change (FC) of the differentially expressed proteins involved in protein refolding for creeping bentgrass (Crenshaw and S11 729‐10) when heat‐stressed samples were compared against control samples at 14 and 28 days (A). The corresponding protein description, including full name and short name for each accession ID, was attached (B).

Regarding ROS scavenging, seven out of 11 identified antioxidant proteins showed upregulation in heat‐stressed Crenshaw at 14 days while there were four out of 11 in heat‐stressed S11 729‐10 (Figure 6A). At the end of the trial (28 days), the number of upregulated proteins decreased to three in Crenshaw. Contrastingly, it was increased to seven in S11 729‐10, with the rest showing no differential expression except superoxide dismutase (Cu‐Zn; Figure 6B). For protein refolding, eight DEPs belonging to HSP families including HSP60, 70, 80 and 90, were identified (Figure 7). They were heat‐induced and mostly showed a continuous increase over time. At 28 days, Hsp70‐Hsp90 organizing protein (HOP), and Heat shock 70 kDa protein BIP1 were uniquely accumulated in S11 729‐10 and Crenshaw, respectively.

### 3.3 Polyubiquitin‐Omics

**FIGURE 8:**
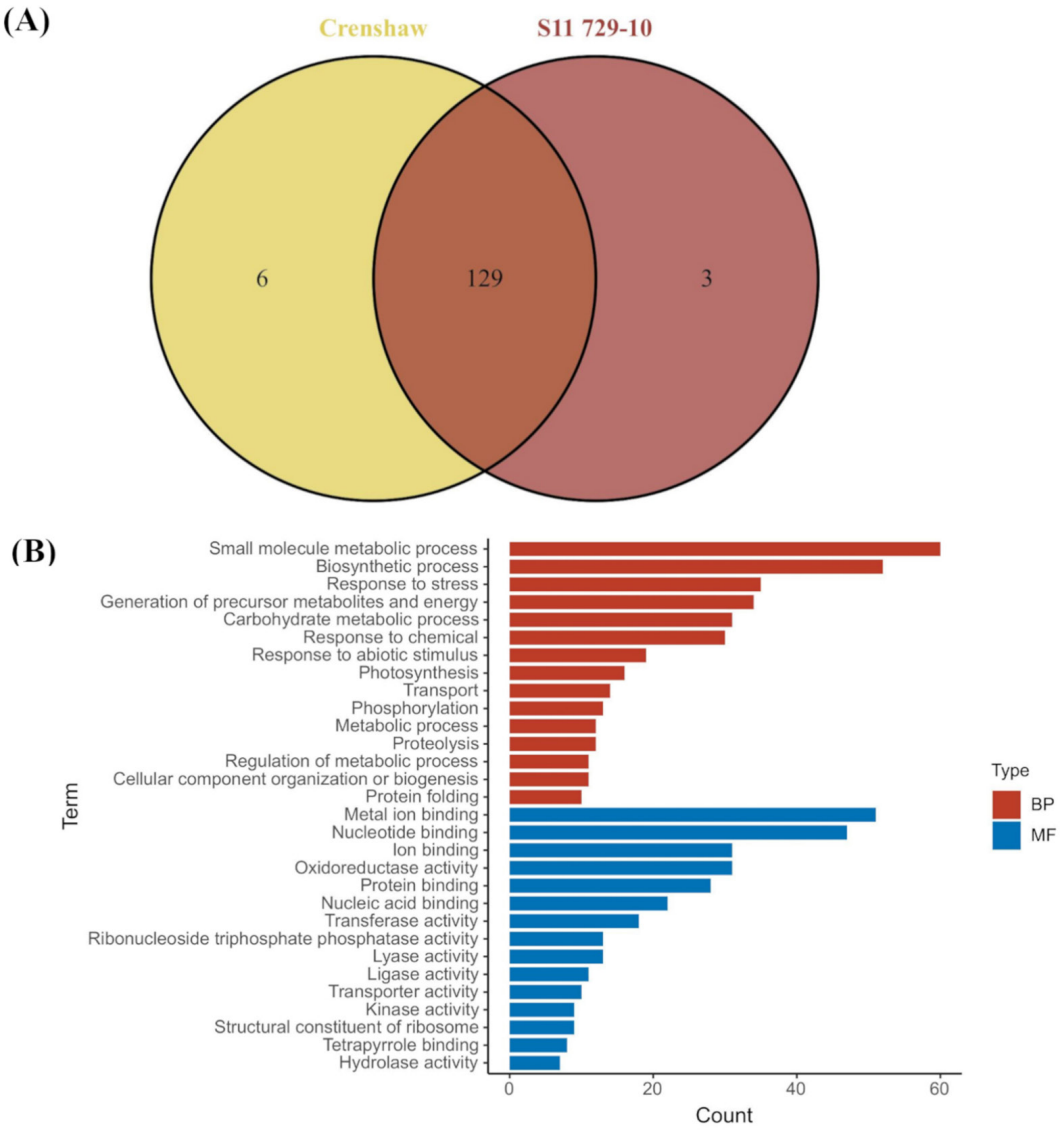
Identified polyubiquitinated proteins of two heat‐stressed creeping bentgrass lines (Crenshaw and S11 729‐10) at 28 days. (A) Venn diagram showing common and unique polyubiquitinated proteins. (B) Bar plot showing Top 30 GO terms for commonly identified polyubiquitinated proteins. Count was the number of polyubiquitinated proteins falling into each GO term. BP, biological process; MF, molecular function.

Out of a total of 138 putative polyubiquitinated proteins identified under heat stress, six were uniquely identified in Crenshaw (2,3‐bisphosphoglycerate‐independent phosphoglycerate mutase [P30792]; 6‐phosphogluconate dehydrogenase, decarboxylating [A0A1D6LJP0]; Cysteine synthase [A0A0E0GTN2]; DNA‐directed RNA polymerase [A0A3L6EAY7]; Ferredoxin‐dependent glutamate synthase (Fragment) [Q08258]; Inositol‐3‐phosphate synthase 1 [O64437]), while only three were identified in S11 729‐10 (Beta‐fructofuranosidase, insoluble isoenzyme 4 [Q5JJV0]; Peroxidase [A0A1D5UL37]; Superoxide dismutase [Cu–Zn] 4AP [P23346]; Figure 8A). A total of 129 polyubiquitinated proteins were identified in both lines. According to GO analysis on these polyubiquitinated proteins, the most represented GO terms for BPs were small molecular metabolic process, biosynthetic process, response to stress, generation of precursor metabolites and energy, carbohydrate metabolic process (Figure 8B). A few other important heat‐responsive processes were also included, such as photosynthesis, proteolysis, and protein binding. Regarding MF, the top GO categories associated with identified polyubiquitinated proteins were metal ion binding, nucleotide binding, ion binding, oxidoreductase binding, as well as protein binding.

Among 138 identified polyubiquitinated proteins, 20 of them were considered to be DEPs when comparing heat‐stressed S11 729‐10 against heat‐stressed Crenshaw (Table 1). Specifically, eight polyubiquitinated DEPs showed a significant downregulation in S11 729‐10, with FC values ranging from 0.2 to 0.6. Among these downregulated polyubiquitinated DEPs, one was identified as an E3 ligase while half were members of the histone family. Contrastingly, 12 polyubiquitinated DEPs were upregulated instead in heat‐stressed S11 729‐10, with FC values ranging from 1.2 to 6.0. The majority of the upregulated polyubiquitinated DEPs were involved in antioxidant defense, chaperone activity and energy metabolism.

**TABLE 1:** List of differentially expressed polyubiquitinated proteins with their accession ID, description, and fold change (FC) when comparing S11 729‐10 against Crenshaw under heat stress at 28 days.

Accession	Description	FC
A0A3B6KQP3	Catalase OS = *Triticum aestivum*	1.2
Q6ZBZ2	Germin‐like protein 8–14 OS = *Oryza sativa* subsp. *japonica*	2.7
A0A3B6PVU5	l‐ascorbate peroxidase OS = *T. aestivum*	2.2
A0A0E0QCR5	Plant heme peroxidase family profile domain‐containing protein OS = *Oryza rufipogon*	5.7
Q67UK9	Probable glutathione S‐transferase DHAR2, chloroplastic OS = *O. sativa* subsp. *japonica*	2.5
Q84Q72	18.1 kDa class I heat shock protein OS = *O. sativa* subsp. *japonica*	3.9
P21569	Peptidyl‐prolyl cis‐trans isomerase OS = *Zea mays*	4.8
P00068	Cytochrome c OS = *T. aestivum*	3.6
Q7FAH2	Glyceraldehyde‐3‐phosphate dehydrogenase 2, cytosolic OS = *O. sativa* subsp. *japonica*	0.6
A0A0E0EN62	Photosystem I iron–sulfur center OS = *Oryza meridionalis*	6.0
A0A0E0HXE6	Pyruvate kinase OS = *Oryza nivara*	5.8
Q8L5C6	Xylanase inhibitor protein 1 OS = *T. aestivum*	0.6
A0A1D6HLU2	Kinesin‐like protein OS = *Z. mays*	1.5
P02276	Histone H2A.2.1 OS = *T. aestivum*	0.3
A2WKT4	Histone H2B.5 OS = *O. sativa* subsp. *indica*	0.2
P68428	Histone H3.2 OS = *T. aestivum*	0.3
P62787	Histone H4 OS = *Z. mays*	0.4
A0A0E0D0N3	RING‐type E3 ubiquitin transferase OS = *Oryza meridionalis*	0.3
C5WVT9	Guanine nucleotide‐binding protein alpha subunit OS = *Sorghum bicolor*	5.9
Q7F8T6	Tricin synthase 2 OS = *O. sativa* subsp. *japonica*	0.3

Abbreviation: OS, organism species.

## 4 Discussion

The three investigated lines showed differential levels of heat tolerance, as reflected by the differences in their overall performance (TQ, green cover). In accordance with the tolerance ranking in previous findings (Fan and Jespersen 2023), S11 729‐10 was the best performer while Crenshaw was a relatively poor performer and S11 675‐02 was intermediate in its responses. Superior overall performance in the more heat‐tolerant S11 729‐10 was associated with its improved physiological responses, including greater cell membrane stability as measured by EL, and healthier photosynthetic status as evaluated by chlorophyll fluorescence traits (ABS/CSm, DIo/ABS, Fv/Fm, ETo/CSm, and REo/CSm). Accumulated ROS triggered by heat stress can attack lipids, resulting in decreased membrane stability with concomitant peroxidation of lipids (Liu and Huang 2000). Hence, EL increases during exposure to stress, but plants with greater thermotolerance typically have lower values of EL (Du et al. 2009; Fan and Jespersen 2023), which corroborates the results found in our study. By monitoring the rise of fluorescence intensity to a maximum at various states, chlorophyll fluorescence parameters can quantify sequential light harvesting processes, including light absorption by PSII antenna (ABS/CSm), energy trapping by PSII reaction centers (Fv/Fm), energy dissipation (DIo/ABS), electrons migration toward intersystem acceptors from PSII (ETo/CSm) as well as electron transport into PSI end acceptors (REo/CSm; Stirbet et al. 2018). This tool is gaining increasing popularity for stress detection in various plant species due to its rapidness, sensitivity, and reliability (Snider et al. 2018; Fan and Jespersen 2023; Fan, Raymer, et al. 2024; Zhou et al. 2024). In our study, at 28 days, heat stress resulted in different extents of decreases in ABS/CSm, Fv/Fm, ETo/CSm, and REo/CSm while concurrent increases in DIo/ABS among lines relative to control. The increase in DIo/ABS highlighted that stress‐induced damage required the leaves to dissipate excess excitation energy instead of utilizing it for photosynthetic processes, thus being negatively correlated with heat tolerance ranking (Chen et al. 2016). Collectively, these changes in fluorescence traits indicated that the injuries to photosynthetic components were widespread in the chloroplast and that these light‐harvesting steps might be concomitantly damaged by heat stress (Fan and Jespersen 2023). Although disruption of photosynthetic metabolism was a common response, S11 729‐10 was able to minimize the damage as evidenced by greater values for ABS/CSm, Fv/Fm, ETo/CSm, and REo/CSm while smaller values for DIo/ABS, compared to the other two lines. Similar findings have been documented in previous literature where more heat‐tolerant cultivars or lines typically exhibited less variation in fluorescence parameters, although photosynthetic components were commonly damaged (Chen et al. 2016; Zhang et al. 2022; Fan and Jespersen 2023).

Proteins are important drivers behind physiological responses. Stress not only triggers differences in physiological performance, but also elicits differential accumulation of proteins involved in those activities correspondingly (Jespersen et al. 2015; Zhao and Hou 2023; Schmitt et al. 2024). Since the most significant differences in terms of physiological traits were detected between S11 729‐10 and Crenshaw as discussed above, analysis of global protein accumulation was performed for these two lines. Differential changes in protein accumulation resulted in clear separation between the two contrasting lines based on PCA analysis, which explained a large proportion of the observed variance. This implied that the difference in heat tolerance between the two lines could be attributed, at least in large part, to these DEPs. Interestingly, more DEPs were detected at 14 d compared to 28 d for both lines. During the early phase of stress, proteomic alterations could occur rapidly and robustly to help plants adapt to stress and maintain cellular homeostasis under challenging conditions. However, when stress treatments are prolonged, plants could fail to maintain stress‐induced homeostasis and enter an exhaustion phase when they became less responsive to the stressor (Kosová et al. 2011). The reduced responsiveness resulted from the depletion of essential resources, such as energy and nutrients, required for defense or repair pathways, as well as the accumulation of damages (Kosová et al. 2011). As a result, there would be a reduction in the number of DEPs compared to the early phase. Additionally, DEPs related to carbohydrate metabolism, antioxidant defense and protein metabolism were major categories identified by both GO term and pathway analysis, which were highly regulated during both time points, particularly, during the later phase of heat stress. Since photosynthesis inhibition, oxidative stress and protein damage are the most common symptoms induced by heat stress, with a large number of identified DEPs associated with these processes as supported by previous literature (Li, Nadeem, et al. 2020; Li, Zeng, et al. 2020) as well as the GO term analysis in our study, we put additional focus on the DEPs related to light reactions, antioxidant defense, and protein refolding and degradation.

### 4.1 DEPs Associated With Photosynthesis

Photosynthesis is the basis of plant growth. It consists of two phases: light reactions and dark reactions. During light reactions, light is absorbed by chlorophyll and then transported along an electron transport chain, leading to the production of ATP and NADPH, which are essential for the subsequent dark reactions. Four major protein complexes are involved in light reactions: PSII, Cytochrome b_6_f, PSI, and ATP synthase (Taiz and Zeiger 2006). Impairment of these complexes can weaken photosynthetic ability. Declines in the abundance of light‐reaction‐related proteins have been documented to be common responses under heat stress in various plant species besides creeping bentgrass (Jespersen et al. 2015), such as *A. thaliana* (Rocco et al. 2013), soybean ( *Glycine max* L.; Ahsan et al. 2010), wheat ( *T. aestivum* ; Li et al. 2017), and rice ( *O. sativa* L.; Han et al. 2009). Nevertheless, more heat‐tolerant plants generally possessed less severe downregulation of these proteins (Xu and Huang 2012; Jespersen et al. 2015; Li et al. 2017). For instance, photosynthesis‐related proteins including ATP‐synthase, cytochrome b6f, and chloroplast oxygen‐evolving enhancer proteins were downregulated in two lines of bentgrass (ColxCB169 and ColxCB190) due to heat stress, but they were decreased later and to a lesser extent in leaves of the more heat‐tolerant bentgrass line ColxCB169 (Jespersen et al. 2015). These corroborate the results found in our study, where heat stress mostly caused downregulation of DEPs involved in the electron transport chain, but the number of downregulated DEPs, as well as the extent of decreases, were both smaller in S11 729‐10 relative to Crenshaw. Furthermore, as heat stress progressed into 28 days, the accumulation of most proteins involved in light reactions was further reduced in Crenshaw, reflecting more severe damage under prolonged stress. However, this was not observed in S11 729‐10. Instead, quite a few proteins showed an unchanged accumulation (Cytochrome b6‐f complex iron–sulfur subunit, PSI assembly protein Ycf4, PSI chlorophyll *a* apoprotein A1/A2, Ferredoxin–NADP reductase, and ATP synthase subunit alpha) or an increased accumulation (Chlorophyll *a‐b* binding protein, PSII 22 kDa protein 1) in stressed S11 729‐10 relative to control conditions at 28 days. Furthermore, these proteins were specifically involved in light absorption by PSII antenna, energy trapping by PSII reaction centers, and energy flux associated with electron transport from intersystem to final PSI acceptors, suggesting better maintenance of sequential photosynthetic component processes in heat‐tolerant S11 729‐10, especially during the later phase of stress. These were consistent with the measurements of chlorophyll fluorescence traits in our study. Healthier light harvesting components, eventually, could lead to greater production of ATP and NADPH, which are an important energy source and reducing agent, respectively, and could impact numerous cellular activities beyond the dark reaction (Taiz and Zeiger 2006).

### 4.2 DEPs Associated With Antioxidant Defense

High temperature accelerates ROS production, resulting in oxidative stress, making enhanced antioxidant capacity one of the most fundamental protective responses (Wang et al. 2024). Increased activity or accumulation of antioxidants, like peroxidase, superoxide dismutase, and ascorbate peroxidase, has been reported previously when plants were exposed to heat stress (Li et al. 2014; Li, Nadeem, et al. 2020; Li, Zeng, et al. 2020). Typically, more heat‐tolerant plants would present stronger antioxidant activity due to greater accumulation of antioxidants, as supported by these studies. In addition to cultivar or line differences, antioxidant capacity was also affected by stress duration. For instance, the activity of superoxide dismutase increased in creeping bentgrass leaves at 18 days of heat stress, but then presented significant declines at 28 and 35 days (Li et al. 2016; Li, Zeng, et al. 2020). The authors highlighted that plants could activate antioxidant defense to acclimate to oxidative damage in response to an earlier phase of heat stress, but antioxidant defense decreased as a consequence of damage accumulation when plants suffered from severe stress at a later phase. Similar findings were confirmed in our study. Specifically, the expression of identified antioxidant proteins was mostly enhanced in Crenshaw at 14 days. Moreover, the responses were stronger compared to S11 729‐10 on the same day, as evidenced by a larger number of upregulated DEPs as well as greater extents of upregulation, indicating that Crenshaw might be experiencing a more severe ROS attack. From 14 to 28 days, the number of upregulated DEPs (catalase, superoxide dismutase [Mn], thioredoxin reductase) decreased for Crenshaw, although the extents of upregulation were enhanced. When stress persisted for longer durations, antioxidant proteins can become denatured and nonfunctional due to accumulated damage from heat stress and ROS, which might explain the fewer upregulated DEPs. In contrast to the decrease for Crenshaw, an increase in the number of upregulated DEPs like catalase, superoxide dismutase [Mn], l‐ascorbate peroxidase, probable l‐ascorbate peroxidase 8, ferritin, peptide methionine sulfoxide reductase A4, and probable glutathione S‐transferase DHAR2 was detected for S11 729‐10 at 28 days relative to 14 days, indicating improved antioxidant defense. Furthermore, at 28 days, antioxidant proteins in S11 729‐10 mostly showed enhanced expression or better maintenance of accumulation compared to the corresponding ones in Crenshaw. This might contribute to the reduced oxidative stress observed in the heat‐tolerant S11 729‐10, as supported by its greater cell membrane stability, as evaluated by EL. Overall, these suggest that Crenshaw experienced more severe oxidative stress during the early phase of heat stress, while S11 729‐10 enhanced its survival by increasing the accumulation of antioxidant proteins during the later phase, thereby leading to improved performance under prolonged stress (Li et al. 2014).

### 4.3 DEPs Associated With Protein Folding and Degradation

The induction of HSPs is a common response to the formation of aberrant proteins induced by heat stress (Parsell and Lindquist 1993). HSPs work by promoting refolding of misfolded proteins, thereby helping maintain proteins' functional conformations. Consistent with previous literature (Wang et al. 2014; Li, Cheng, et al. 2020; Li, Nadeem, et al. 2020; Li, Zeng, et al. 2020), enhanced accumulation of HSPs of different sizes, such as HSP60, HSP70, and HSP80, were detected under temperature elevation in both lines. Plus, the level of upregulation for these HSPs continued to rise from 14 to 28 days, possibly suggesting increased accumulation of damaged proteins. Particularly, Chaperonin CPN60‐1 belonging to the HSP60 family was upregulated prominently, by 9.4 and 9.5‐fold in stressed Crenshaw and S11 729‐10, respectively, at 28 days. This dramatic upregulation might suggest its crucial role in protein repair in both lines. Despite the common induction, line‐specific protein induction was also observed. For example, HOP was uniquely induced in S11 729‐10 while BIP1 was only upregulated in Crenshaw at 28 days. HOP is a family of cytosolic cochaperones whose role in thermotolerance is deeply analyzed in other eukaryotes, but is largely unexplored in plants with the exception of a few studies (Toribio et al. 2020). In Arabidopsis, HOP3 was highly induced in response to temperature elevation and HOP‐overexpressing plants displayed enhanced tolerance to heat (Fernández‐Bautista et al. 2018; Wang et al. 2023). Contrastingly, the HOP mutants rendered Arabidopsis sensitive to heat stress with an unusually high accumulation of insoluble and ubiquitinated proteins, which underscores the crucial role of HOP in protein quality control under heat (Fernández‐Bautista et al. 2018). Unlike the significant gap in the study of HOP in heat stress, more efforts have been made to advance the understanding of BIP in thermotolerance. BIP expression was reported to be upregulated in response to temperature elevation in various plants (Jung et al. 2013; Zhang, Zhao, et al. 2015; Yang et al. 2016; Wang, Niu, et al. 2017). In pepper ( *Capsicum annuum* L.), a BIP‐overexpression line displayed improved heat tolerance with reduced oxidative damage, as manifested by lower contents of malondialdehyde and H_2_O_2_, while the silencing of the BIP1 gene resulted in more severe injury symptoms, rendering it susceptible to heat stress (Wang, Niu, et al. 2017). Similar results were also found in Arabidopsis (Yang et al. 2016), suggesting the protective role of BIP1 against heat stress. The unique induction of these HSPs might indicate the activation of different defense pathways and contribute to the contrasting heat tolerance between creeping bentgrass lines S11 729‐10 and Crenshaw.

In addition to being repaired, another fate for damaged proteins is to be degraded through a proteolytic machinery like proteases and the UPS, as described earlier. Lower proteolysis activity typically corresponded to less severe protein damage and higher protein content, in turn associated with greater thermotolerance (Chauhan et al. 2009; Rossi and Huang 2023). In our study, two proteolysis‐related proteins were identified to be differentially expressed, which were proteasome subunit beta and endopeptidase Clp (Table S2–S5). The former is an integral component of the UPS while the latter belongs to the category of serine proteases (Roberts et al. 2012; Stone 2019). Intriguingly, no matter at 14 or at 28 days, both proteasome subunit beta and endopeptidase Clp were downregulated to greater extents in S11 729‐10 than in Crenshaw, possibly suggesting lower proteolytic activity and slower protein degradation in S11 729‐10, as enzyme concentration typically correlates positively with catalytic activity (Taiz and Zeiger 2006). This could be associated with less severe downregulation of proteins involved in important pathways, like the aforementioned light reaction of photosynthesis, conferring greater thermotolerance in heat‐tolerant S11 729‐10.

### 4.4 Differentially and Uniquely Expressed Polyubiquitinated Proteins

Using polyubiquitin‐omics, a number of polyubiquitinated proteins were identified to be associated with various important cellular activities, including photosynthesis, protein folding, proteolysis, transport, signal transduction, and redox homeostasis, as supported by GO term analysis. In addition to line‐unique expression, differential accumulation of polyubiquitinated proteins was detected in S11 729‐10 relative to Crenshaw in response to temperature elevation, with most of these proteins enriched in antioxidant defense, energy metabolism, and protein metabolism. Similar attempts on the identification of substrate proteins targeted by the UPS pathway have also been made previously under various environmental conditions (Xu et al. 2024). For instance, enhanced ubiquitination was found when rice roots were exposed to heat stress, with the majority of polyubiquitinated proteins being associated with sucrose and starch metabolism, as well as the ribosomal system (Ying et al. 2023). In the case of salt stress, greater upregulation of ubiquitin‐modified HSP81‐1 and aldehyde oxidase 3 was seen in rice lines—TNG67 and SA0604, indicating more severe protein degradation via the UPS, which might contribute to the inferior salt tolerance in TNG67 and SA0604 compared to the more tolerant SM75 (Liu et al. 2012).

Among those differentially and uniquely expressed polyubiquitinated proteins identified in our study, antioxidant proteins were either significantly upregulated (Catalase, Germin‐like protein 8–14, l‐ascorbate peroxidase, Plant heme peroxidase family profile domain‐containing protein, Probable glutathione S‐transferase DHAR2) or uniquely induced (Peroxidase, Superoxide dismutase [Cu‐Zn] 4AP) in S11 729‐10 compared to Crenshaw, potentially suggesting a faster turnover. Heat stress leads to accumulation of damaged proteins and an elevated need for ROS detoxication. To meet this demand, plants might rapidly degrade antioxidant proteins that were denatured or oxidized through the UPS, while synthesizing more new antioxidant proteins for replacement, contributing to reduced oxidative damage and thereby improved tolerance (Li et al. 2022; Fan, Xu, et al. 2024). This is consistent with the enhanced expression of several antioxidant proteins in S11 729‐10 (Catalase, l‐ascorbate peroxidase, Plant heme peroxidase family profile domain‐containing protein, Probable glutathione S‐transferase DHAR2) when heat‐stressed samples were compared against control ones at 28 days according to the gel‐free proteomics results (Figure 6). Similarly, greater upregulation of polyubiquitinated proteins involved in energy metabolism (Cytochrome c, Photosystem I iron–sulfur center, Pyruvate kinase), chaperone activity (18.1 kDa Class I HSP, Peptidyl‐prolyl cis‐trans isomerase), and transport (Kinesin‐like protein) was observed in S11 729‐10 than Crenshaw. The faster turnover of these polyubiquitinated proteins involved in energy metabolism, chaperone activity, and transport might enable more efficient ATP production, protein refolding of damaged proteins, as well as transport of key molecules involved in stress responses, respectively, leading to improved tolerance in S11 729‐10. These results are in accordance with previous research where higher degradation rates of HSPs, antioxidant proteins (catalases and peroxidases), and photorespiration‐related proteins were detected when *A. thaliana* seedlings were exposed to elevated temperatures (Fan, Xu, et al. 2024). The authors proposed that the faster turnover might represent an important adaptive mechanism to maintain cellular homeostasis and improve plant survival under stressful conditions. In contrast to the upregulation mostly observed, polyubiquitinated E3 ligase showed a significant downregulation in heat‐stressed S11 729‐10 compared to heat‐stressed Crenshaw, potentially indicating greater stability. This is further supported by the gel‐free proteomics results, which found that E3 ligase levels were better maintained in S11 729‐10 compared to Crenshaw under heat stress, relative to control conditions. Given the role of E3 ligase in tagging substrate proteins for degradation via the UPS, better maintenance of E3 ligase levels enables more effective removal of heat‐induced damaged proteins, reducing cellular toxicity and preserving protein quality (Shu and Yang 2017). On the other hand, E3 ligases are involved in modulating key stress‐responsive proteins, including transcription factors and signaling molecules (Shu and Yang 2017). By ensuring that these regulatory proteins are activated or degraded as needed, better maintenance of E3 ligase levels allows plants to adjust more rapidly and robustly to heat stress. Taken together, the accumulation of polyubiquitinated proteins in plants during heat stress is a complex process, and changes in the levels of proteins and their polyubiquitinated forms are not always synchronized, despite that ubiquitination typically leads to protein degradation.

## 5 Conclusions

Gel‐free proteomics was applied to heat‐stressed creeping bentgrass to reveal the change in proteome profile, determining differential physiological performance among the three investigated lines. It showed that some common metabolic processes, like photosynthesis, antioxidant defense, and protein refolding, could be responsible for regulating heat tolerance in contrasting lines. Heat‐tolerant S11 729‐10 was able to maintain less severe downregulation of the proteins involved in the light reactions of photosynthesis while enhancing the upregulation of antioxidant proteins, particularly during the later phase of stress. These contributed to improved physiological responses, including greater cell membrane stability as well as healthier light‐harvesting components, eventually leading to higher overall tolerance levels in S11 729‐10. For the first time, polyubiquitin‐omics analysis was applied to turfgrass research, revealing differentially or uniquely expressed polyubiquitinated proteins in S11 729‐10, with enrichment in antioxidant defense, energy production, and protein metabolism. Notably, the faster turnover of key polyubiquitinated antioxidant proteins in S11 729‐10 likely represents a critical mechanism for protecting against oxidative damage and enhancing tolerance under prolonged heat stress. Our findings suggest the power of gel‐free proteomics and polyubiquitin‐omics in improving the understanding of global protein accumulation and degradation through the UPS, as well as their associated physiological responses. The stress‐related traits or proteins identified in this study could be utilized for the development of new cultivars with enhanced thermotolerance to help plants cope with climate change. Further studies are needed to gain a more complete picture of how protein metabolism is regulated at multiple levels.

## Author Contributions

Q.F. was involved in the conceptualization, methodology, formal analysis of the experiments, data curation, and writing and editing. C.‐W.C. was involved in the methodology, reviewing, and editing the manuscript. D.J. was involved in the conceptualization of experiments, resource, and funding acquisition, supervision, and the reviewing and editing of the manuscript.

## Conflicts of Interest

The authors declare no conflicts of interest.

## Supporting information


**Data S1:** ppl70568‐sup‐0001‐Supinfo.pdf.

## Data Availability

The data underlying this article are available in the article and in its Supplementary Data published online. The mass spectrometry proteomics data have been deposited to the ProteomeXchange Consortium via the PRIDE partner repository with the dataset identifier PXD060809 and 10.6019/PXD060809. The following Supplementary Data is available at *Physiologia Plantarum* online.
