# Supplementary material for: Protein Metabolism Underlying Heat Tolerance in Contrasting Creeping Bentgrass Lines: Insights From Gel‐Free Proteomics and Polyubiquitin‐Omics
Source: Physiol Plant. 2025 Oct 10;177(5):e70568. doi: 10.1111/ppl.70568 (PMC12513134; doi:10.1111/ppl.70568)
Supplement: Supplementary file 1 — Data S1: ppl70568‐sup‐0001‐Supinfo.pdf. [file PPL-177-e70568-s001.pdf]

Table S1 ANOVA results for heat stress trial of creeping bentgrass

Parameter	*P* value					
	Control			Heat stress		
	Line	Date	Line × Date	Line	Date	Line × Date
TQ	0.354	0.151	0.130	<0.001	<0.001	<0.001
Green cover	0.081	0.003	0.359	<0.001	<0.001	<0.001
EL	0.012	0.543	0.118	<0.001	<0.001	<0.001
Fv/Fm	0.332	0.014	0.578	0.011	<0.001	0.016
ABS/CSm	0.151	-	-	<0.001	-	-
DIo/ABS	0.146	-	-	0.021	-	-
ETo/CSm	0.468	-	-	0.004	-	-
REo/CSm	0.194	-	-	0.003	-	-

TQ, turf quality; EL, electrolyte leakage; Fv/Fm, quantum efficiency of energy flux trapped by photosystem II (PSII) photochemistry; ABS/CSm, absorbed energy flux per cross section; DIo/ABS, quantum efficiency of energy dissipation in PSII antenna; ETo/CSm, the energy flux associated with electron transport from quinone A to intersystem electron acceptors such as plastoquinone pool per cross section; REo/ CSm, the energy flux associated with electron transport from intersystem electron acceptors to final photosystem I acceptors per cross section

Table S2 Change in visual turf quality rating for creeping bentgrass lines over time under control (20/15°C day/night) and heat stress (38/33°C day/night) conditions

Lines	Control					Heat				
	**0**	**7**	**14**	**21**	**28**	**0**	**7**	**14**	**21**	**28**
Crenshaw	9.0	8.9	9.0	9.0	9.0	9.0	8.2b	7.6b	6.9b	4.0b
S11 675-02	8.9	9.0	9.0	9.0	9.0	9.0	8.5a	7.9a	6.8b	5.9a
S11 729-10	9.0	9.0	9.0	9.0	9.0	9.0	8.5a	8.0a	7.6a	7.2a
LSD	ns	ns	ns	ns	ns	ns	0.22	0.25	0.52	1.6

Note: values followed by a common lowercase letter within each column indicate no significant difference among various lines at $p = 0.05$; ns, not significant

Table S3 Change in green cover for creeping bentgrass lines over time under control (20/15°C day/night) and heat stress (38/33°C day/night) conditions

**Lines**	**Control**					**Heat**				
	**0**	**7**	**14**	**21**	**28**	**0**	**7**	**14**	**21**	**28**
Crenshaw	97.8a	95.4	95.4	91.2	98.5a	96.6	87.9	87.9	61.2b	22.0b
S11 675-02	94.5b	96.1	96.1	89.4	91.9b	95.3	93.3	93.3	67.3ab	46.6a
S11 729-10	98.2a	93.7	93.6	89.4	95.0ab	95.0	93.0	93.0	78.3a	60.9a
LSD	2.2	ns	ns	ns	4.6	ns	ns	ns	11.9	18.5

Note: values followed by a common lowercase letter within each column indicate no significant difference among various lines at $p = 0.05$; ns, not significant

Table S4 Change in electrolyte leakage for creeping bentgrass lines over time under control (20/15°C day/night) and heat stress (38/33°C day/night) conditions

Lines	Control					Heat				
	**0**	**7**	**14**	**21**	**28**	**0**	**7**	**14**	**21**	**28**
Crenshaw	15.4	15.8	14.1	13.2b	13.2b	14.7	24.9a	30.6	64.2a	73.7a
S11 675-02	15.1	16.7	20.8	15.7b	15.7b	15.4	22.1ab	27.1	46.9b	58.3b
S11 729-10	16.2	15.6	14.8	20.9a	20.9a	15.8	18.9b	25.3	35.8b	44.6c
LSD	ns	ns	ns	4.0	4.0	ns	4.3	ns	12.5	9.1

Note: values followed by a common lowercase letter within each column indicate no significant difference among various lines at $p = 0.05$; ns, not significant

Table S5 Change in Fv/Fm for creeping bentgrass lines over time under control (20/15°C day/night) and heat stress (38/33°C day/night) conditions

**Lines**	**Control**					**Heat**				
	**0**	**7**	**14**	**21**	**28**	**0**	**7**	**14**	**21**	**28**
Crenshaw	0.82	0.82	0.80	0.81	0.82	0.80	0.75	0.75	0.694b	0.48b
S11 675-02	0.82	0.83	0.81	0.81	0.83	0.81	0.77	0.75	0.719ab	0.61ab
S11 729-10	0.82	0.82	0.81	0.81	0.82	0.82	0.75	0.74	0.723a	0.69a
LSD	ns	ns	ns	ns	ns	ns	ns	ns	0.027	0.14

Note: values followed by a common lowercase letter within each column indicate no significant difference among various lines at $p = 0.05$; ns, not significant; Fv/Fm, quantum efficiency of energy flux trapped by photosystem II photochemistry.

Table S6 List of differentially expressed proteins with their accession ID, description and log2 fold change (logFC) when comparing heat stress condition against control condition for Crenshaw at 14 d

Accession	Description	logFC
P30792	2,3-bisphosphoglycerate-independent phosphoglycerate mutase OS=Zea mays	-0.52
W5EP13	2-carboxy-D-arabinitol-1-phosphatase OS=Triticum aestivum	1.46
Q6AVG6	4-hydroxy-3-methylbut-2-enyl diphosphate reductase, chloroplastic OS=Oryza sativa subsp. japonica	0.46
A0A2S3IJL2	5-methyltetrahydropteroyltriglutamate--homocysteine S-methyltransferase OS=Panicum hallii	-1.55
A0A2T7DTW1	AAA+ ATPase domain-containing protein OS=Panicum hallii var. hallii	-1.71
A0A3B6NJT0	Acetyltransferase component of pyruvate dehydrogenase complex OS=Triticum aestivum	0.81
P32112	Adenosylhomocysteinase OS=Triticum aestivum	-0.97
A0A3B6PR10	ADP/ATP translocase OS=Triticum aestivum	-0.37
P34106	Alanine aminotransferase 2 OS=Panicum miliaceum	0.82
A0A1D6HR58	alanine transaminase OS=Zea mays	-1.3
Q0DWH1	Alcohol dehydrogenase class-3 OS=Oryza sativa subsp. japonica	0.67
A0A1B6QHW4	Alpha-1,4 glucan phosphorylase OS=Sorghum bicolor	-0.86
Q9FXT4	Alpha-galactosidase OS=Oryza sativa subsp. japonica	-1.57
A0A453NS22	Aminopeptidase OS=Aegilops tauschii subsp. strangulata	0.34
Q9XJ29	Anthranilate synthase alpha subunit 2, chloroplastic OS=Oryza sativa subsp. japonica	0.46
B8AU84	Arginase 1, mitochondrial OS=Oryza sativa subsp. indica	2.42
P37833	Aspartate aminotransferase, cytoplasmic OS=Oryza sativa subsp. japonica	-1.1
A0A1B6PGM0	aspartate carbamoyltransferase OS=Sorghum bicolor	1.37
A0A3B5ZZW5	assimilatory sulfite reductase (ferredoxin) OS=Triticum aestivum	-1.19
A0A2L0VAS4	ATP synthase subunit alpha, chloroplastic OS=Lamarckia aurea	-1.22
A0A2L0VAT4	ATP synthase subunit beta, chloroplastic OS=Lamarckia aurea	-1.26
P0C1M0	ATP synthase subunit gamma, chloroplastic OS=Zea mays	-0.94
A0A317YBF8	ATP-dependent DNA helicase OS=Zea mays	1.73
P16098	Beta-amylase OS=Hordeum vulgare	-1.91
A0A3L6FGN4	carbamoyl-phosphate synthase (glutamine-hydrolyzing) OS=Zea mays	0.7

B9EXM2	Carbamoyl-phosphate synthase large chain, chloroplastic OS=Oryza sativa subsp. japonica	0.76
A0A3B6KQP3	Catalase OS=Triticum aestivum	1.31
A0A1W0VZF1	CBM20 domain-containing protein OS=Sorghum bicolor	3.11
P29185	Chaperonin CPN60-1, mitochondrial OS=Zea mays	2.35
A2XJ35	Chlorophyll a-b binding protein, chloroplastic OS=Oryza sativa subsp. indica	-0.29
A0A0E0GTN2	Cysteine synthase OS=Oryza nivara	-2.28
A0A024BKI8	Cytochrome b559 subunit alpha OS=Cenchrus americanus	-0.63
A0A3G1AT48	Cytochrome b6 OS=Bromus vulgaris	-1.16
Q7X9A6	Cytochrome b6-f complex iron-sulfur subunit, chloroplastic OS=Triticum aestivum	-0.83
A0A0E0E626	Delta-aminolevulinic acid dehydratase OS=Oryza meridionalis	0.81
A0A0E0JJD7	Endopeptidase Clp OS=Oryza punctata	-1.16
P36183	Endoplasmin homolog OS=Hordeum vulgare	2.47
A4KAG8	Ent-isokaur-15-ene synthase OS=Oryza sativa subsp. japonica	-0.57
Q6Z2Z4	Eukaryotic initiation factor 4A-3 OS=Oryza sativa subsp. japonica	-0.6
Q69RJ0	Ferredoxin-dependent glutamate synthase, chloroplastic OS=Oryza sativa subsp. japonica	-0.73
P41344	Ferredoxin--NADP reductase, leaf isozyme 1, chloroplastic OS=Oryza sativa subsp. japonica	-1.59
Q6ZFJ3	Ferredoxin--NADP reductase, leaf isozyme 2, chloroplastic OS=Oryza sativa subsp. japonica	-1.88
P41345	Ferredoxin--NADP reductase, root isozyme, chloroplastic OS=Oryza sativa subsp. japonica	-1.1
A0A0E0N8J2	ferredoxin--NADP(+) reductase OS=Oryza rufipogon	-1.32
Q42997	Ferredoxin--nitrite reductase, chloroplastic OS=Oryza sativa subsp. japonica	-2.05
A0A1D5YQ12	Ferritin OS=Triticum aestivum	2.06
Q84N28	Flavone O-methyltransferase 1 OS=Triticum aestivum	-1.22
A0A4V6DBK4	Formate dehydrogenase, mitochondrial OS=Setaria viridis	0.59
Q0JGZ6	Fructokinase-1 OS=Oryza sativa subsp. japonica	-0.59
Q0J8G4	Fructokinase-2 OS=Oryza sativa subsp. japonica	-1.38
A0A2K2DTT5	fructose-bisphosphatase OS=Brachypodium distachyon	-0.45
Q40677	Fructose-bisphosphate aldolase, chloroplastic OS=Oryza sativa subsp. japonica	0.95

Q6AVT2	Glucose-1-phosphate adenylyltransferase large subunit 1, chloroplastic/amyloplastic OS=Oryza sativa subsp. japonica	-0.98
P55239	Glucose-1-phosphate adenylyltransferase large subunit 2 (Fragment) OS=Hordeum vulgare	-1
A0A3B6KSU0	Glucose-1-phosphate adenylyltransferase OS=Triticum aestivum	-0.72
A0A0E0CZR5	Glucose-6-phosphate 1-dehydrogenase OS=Oryza meridionalis	1.09
Q33E23	Glutamate dehydrogenase 2, mitochondrial OS=Oryza sativa subsp. japonica	-0.99
I1HQF1	glutamate synthase (NADH) OS=Brachypodium distachyon	-2.22
P13564	Glutamine synthetase leaf isozyme, chloroplastic OS=Hordeum vulgare	0.37
P14655	Glutamine synthetase, chloroplastic OS=Oryza sativa subsp. japonica	0.63
A0A3B6JM67	Glutathione reductase OS=Triticum aestivum	-0.62
P08735	Glyceraldehyde-3-phosphate dehydrogenase 1, cytosolic OS=Zea mays	0.62
A0A077S2R7	Glycine cleavage system P protein OS=Triticum aestivum	0.34
Q10CE4	Glycolate oxidase 1 OS=Oryza sativa subsp. japonica	-0.7
B8B7C5	Glycolate oxidase 5 OS=Oryza sativa subsp. indica	-0.35
A0A0P0VLJ4	GrpE protein homolog OS=Oryza sativa subsp. japonica	1.32
C5WVT9	Guanine nucleotide-binding protein alpha subunit OS=Sorghum bicolor	-0.61
Q0J4P2	Heat shock protein 81-1 OS=Oryza sativa subsp. japonica	1.24
F8RP11	Hsp70-Hsp90 organizing protein OS=Triticum aestivum	1.51
O64437	Inositol-3-phosphate synthase 1 OS=Oryza sativa subsp. japonica	-0.79
P17788	Large ribosomal subunit protein uL2cz/uL2cy OS=Zea mays	-0.87
Q10N21	L-ascorbate peroxidase 1, cytosolic OS=Oryza sativa subsp. japonica	1.55
Q9FE01	L-ascorbate peroxidase 2, cytosolic OS=Oryza sativa subsp. japonica	0.61
A0A3B6PVU5	L-ascorbate peroxidase OS=Triticum aestivum	1.2
Q6K669	Leucine aminopeptidase 2, chloroplastic OS=Oryza sativa subsp. japonica	-0.32
P24067	Luminal-binding protein 2 OS=Zea mays	1.38
A0A0Q3LQ58	Malate dehydrogenase OS=Brachypodium distachyon	0.5
Q9SE94	Methylenetetrahydrofolate reductase (NADH) 1 OS=Zea mays	-0.68
A0A0E0EWM9	Mevalonate kinase OS=Oryza meridionalis	0.75
A0A0E0D501	Multifunctional fusion protein OS=Oryza meridionalis	0.3
A0A1E5VRY2	N-acyl-aliphatic-L-amino acid amidohydrolase OS=Dichanthelium oligosanthes	0.87

A6N0M9	Nucleoside diphosphate kinase 1 OS=Oryza sativa subsp. indica	0.44
B4FK49	Nucleoside diphosphate kinase 1 OS=Zea mays	-0.94
B8AW64	Nucleosome assembly protein 1;2 OS=Oryza sativa subsp. indica	-1.21
A0A0Q3RM53	ornithine aminotransferase OS=Brachypodium distachyon	3.01
A0A3B6LEJ3	Pectinesterase OS=Triticum aestivum	-2.54
Q336R9	Peptide methionine sulfoxide reductase A4, chloroplastic OS=Oryza sativa subsp. japonica	0.85
A0A0D9UZT5	Peptidyl-prolyl cis-trans isomerase OS=Leersia perrieri	-3.62
P27337	Peroxidase 1 OS=Hordeum vulgare	-1.48
A0A453APJ3	Peroxidase OS=Aegilops tauschii subsp. strangulata	-1.64
Q9FR35	Peroxiredoxin-2C OS=Oryza sativa subsp. japonica	-1.08
P29195	Phosphoenolpyruvate carboxylase 1 OS=Sorghum bicolor	0.35
A0A0Q3JDL9	phosphoglucomutase (alpha-D-glucose-1,6-bisphosphate-dependent) OS=Brachypodium distachyon	-2.16
Q43007	Phospholipase D alpha 1 OS=Oryza sativa subsp. japonica	-0.42
A0A3B6HT18	Phosphomannomutase OS=Triticum aestivum	-1.53
A0A220IHK0	Photosystem I iron-sulfur center OS=Aegilops uniaristata	-0.6
A0A2U9DRJ5	Photosystem I P700 chlorophyll a apoprotein A1 OS=Campeiostachys nutans	-1.16
A0A4P8F6B8	Photosystem I P700 chlorophyll a apoprotein A2 OS=Triodia mallota	-0.76
Q943K1	Photosystem II 22 kDa protein 1, chloroplastic OS=Oryza sativa subsp. japonica	-0.56
Q0J8R9	Photosystem II 22 kDa protein 2, chloroplastic OS=Oryza sativa subsp. japonica	-0.92
A0A218LWN1	Photosystem II CP43 reaction center protein OS=Triticum monococcum	-0.7
A0A0S1S3S5	Photosystem II D2 protein OS=Stipa lipskyi	-1.02
A0A5P8FSR9	Photosystem II protein D1 OS=Bambusa variostriata	-1.2
A0A0E0QCR5	Plant heme peroxidase family profile domain-containing protein OS=Oryza rufipogon	-1.79
O64411	Polyamine oxidase 1 OS=Zea mays	-2.21
Q2QP54	Polyprotein of EF-Ts, chloroplastic OS=Oryza sativa subsp. japonica	-1.56

Q6H6D2	Porphobilinogen deaminase, chloroplastic OS=Oryza sativa subsp. japonica	-0.58
A0A0E0NUQ9	Potassium transporter OS=Oryza rufipogon	-1.23
Q69SV0	Probable L-ascorbate peroxidase 8, chloroplastic OS=Oryza sativa subsp. japonica	1.41
P80607	Probable UDP-arabinopyranose mutase 1 OS=Zea mays	-0.94
O22655	Profilin-4 OS=Zea mays	-0.82
A0A3B6RCJ8	Proteasome subunit beta OS=Triticum aestivum	-0.62
Q2RAK2	Pyruvate kinase 1, cytosolic OS=Oryza sativa subsp. japonica	0.36
A0A0E0HXE6	Pyruvate kinase OS=Oryza nivara	-1.23
A0A2S3ID39	Receptor-like serine/threonine-protein kinase OS=Panicum hallii	-0.93
A0A2T7CH14	Ribulose-phosphate 3-epimerase OS=Panicum hallii var. hallii	-1.23
A0A0E0D0N3	RING-type E3 ubiquitin transferase OS=Oryza meridionalis	-0.58
A0A077RWS5	S-adenosylmethionine synthase OS=Triticum aestivum	-1.28
P46285	Sedoheptulose-1,7-bisphosphatase, chloroplastic OS=Triticum aestivum	-1.64
P49027	Small ribosomal subunit protein RACK1z OS=Oryza sativa subsp. japonica	-1.37
Q6YXW6	Sucrose-phosphatase 2 OS=Oryza sativa subsp. japonica	1.3
A0A3B6RMJ1	Thiamine thiazole synthase, chloroplastic OS=Triticum aestivum	-2.05
A0A0Q3NCX3	Thioredoxin reductase OS=Brachypodium distachyon	0.69
Q7SIC9	Transketolase, chloroplastic OS=Zea mays	-1.22
P46225	Triosephosphate isomerase, chloroplastic OS=Secale cereale	-1.05
Q9ZRB0	Tubulin beta-3 chain OS=Triticum aestivum	2.1
Q8H8T0	UDP-arabinopyranose mutase 1 OS=Oryza sativa subsp. japonica	-1.03
Q6Z7B0	Heat shock 70 kDa protein BIP1 OS=Oryza sativa subsp. japonica	1.36
Q6Z058	Heat shock 70 kDa protein BIP5 OS=Oryza sativa subsp. japonica	1.45
A0A0E0PRJ8	very-long-chain 3-oxoacyl-CoA synthase OS=Oryza rufipogon	1.33
P49087	V-type proton ATPase catalytic subunit A (Fragment) OS=Zea mays	-0.28

Table S7 List of differentially expressed proteins with their accession ID, description and log2 fold change (logFC) when comparing heat stress condition against control condition for Crenshaw at 28 d

Accession	Description	logFC
W5EP13	2-carboxy-D-arabinitol-1-phosphatase OS=Triticum aestivum	2.76
A0A2S3IJL2	5-methyltetrahydropteroyltriglutamate--homocysteine S-methyltransferase OS=Panicum hallii	-1.22
A0A2T7DTW1	AAA+ ATPase domain-containing protein OS=Panicum hallii var. hallii	-2.03
A0A3B6NJT0	Acetyltransferase component of pyruvate dehydrogenase complex OS=Triticum aestivum	1.9
A0A3B6PR10	ADP/ATP translocase OS=Triticum aestivum	0.62
P34106	Alanine aminotransferase 2 OS=Panicum miliaceum	2.14
A0A1D6HR58	alanine transaminase OS=Zea mays	-1.21
Q0DWH1	Alcohol dehydrogenase class-3 OS=Oryza sativa subsp. japonica	0.94
Q9FXT4	Alpha-galactosidase OS=Oryza sativa subsp. japonica	-1.63
A0A0E0L2W9	Amidophosphoribosyltransferase OS=Oryza punctata	0.7
B8AU84	Arginase 1, mitochondrial OS=Oryza sativa subsp. indica	2.84
A0A1B6PGM0	aspartate carbamoyltransferase OS=Sorghum bicolor	1.36
A0A3B5ZZW5	assimilatory sulfite reductase (ferredoxin) OS=Triticum aestivum	-1.69
A0A2L0VAS4	ATP synthase subunit alpha, chloroplastic OS=Lamarckia aurea	-1.93
A0A2L0VAT4	ATP synthase subunit beta, chloroplastic OS=Lamarckia aurea	-1.74
P0C1M0	ATP synthase subunit gamma, chloroplastic OS=Zea mays	-1.71
A0A317YBF8	ATP-dependent DNA helicase OS=Zea mays	3.26
B9EXM2	Carbamoyl-phosphate synthase large chain, chloroplastic OS=Oryza sativa subsp. japonica	2.1
A0A3B6KQP3	Catalase OS=Triticum aestivum	1.71
A0A1W0VZF1	CBM20 domain-containing protein OS=Sorghum bicolor	4.04
P29185	Chaperonin CPN60-1, mitochondrial OS=Zea mays	3.23
A0A0E0GTN2	Cysteine synthase OS=Oryza nivara	-0.9
Q7X9A6	Cytochrome b6-f complex iron-sulfur subunit, chloroplastic OS=Triticum aestivum	-1.14
A0A0E0E626	Delta-aminolevulinic acid dehydratase OS=Oryza meridionalis	1.19
P36183	Endoplasmin homolog OS=Hordeum vulgare	2.38
Q69RJ0	Ferredoxin-dependent glutamate synthase, chloroplastic OS=Oryza sativa subsp. japonica	-1.1
P41344	Ferredoxin--NADP reductase, leaf isozyme 1, chloroplastic OS=Oryza sativa subsp. japonica	-1.98
Q6ZFJ3	Ferredoxin--NADP reductase, leaf isozyme 2, chloroplastic OS=Oryza sativa subsp. japonica	-2.56
P41345	Ferredoxin--NADP reductase, root isozyme, chloroplastic OS=Oryza sativa subsp. japonica	-1.32
A0A0E0N8J2	ferredoxin--NADP(+) reductase OS=Oryza rufipogon	-1.78

Q42997	Ferredoxin--nitrite reductase, chloroplastic OS=Oryza sativa subsp. japonica	-1.83
Q84N28	Flavone O-methyltransferase 1 OS=Triticum aestivum	-0.99
A0A4V6DBK4	Formate dehydrogenase, mitochondrial OS=Setaria viridis	2.39
Q40677	Fructose-bisphosphate aldolase, chloroplastic OS=Oryza sativa subsp. japonica	1.33
Q6AVT2	Glucose-1-phosphate adenylyltransferase large subunit 1, chloroplastic/amyloplastic OS=Oryza sativa subsp. japonica	-0.88
A0A3B6KSU0	Glucose-1-phosphate adenylyltransferase OS=Triticum aestivum	-0.95
I1HQF1	glutamate synthase (NADH) OS=Brachypodium distachyon	-2.01
P13564	Glutamine synthetase leaf isozyme, chloroplastic	1.24
P38562	Glutamine synthetase root isozyme 4 OS=Zea mays	-1.16
P14655	Glutamine synthetase, chloroplastic OS=Oryza sativa subsp. japonica	0.88
Q10CE4	Glycolate oxidase 1 OS=Oryza sativa subsp. japonica	-1.2
B8B7C5	Glycolate oxidase 5 OS=Oryza sativa subsp. indica	-0.82
A0A0P0VLJ4	GrpE protein homolog OS=Oryza sativa subsp. japonica	1.18
Q0J4P2	Heat shock protein 81-1 OS=Oryza sativa subsp. japonica	1.85
O64437	Inositol-3-phosphate synthase 1 OS=Oryza sativa subsp. japonica	-2.22
P17788	Large ribosomal subunit protein uL2cz/uL2cy OS=Zea mays	-1.67
P24067	Luminal-binding protein 2 OS=Zea mays	2.01
Q9SE94	Methylenetetrahydrofolate reductase (NADH) 1 OS=Zea mays	0.87
A0A0D9UZT5	Peptidyl-prolyl cis-trans isomerase OS=Leersia perrieri	-3.7
A0A453APJ3	Peroxidase OS=Aegilops tauschii subsp. strangulata	-2.13
P29195	Phosphoenolpyruvate carboxylase 1 OS=Sorghum bicolor	0.93
A0A0Q3JDL9	phosphoglucomutase (alpha-D-glucose-1,6-bisphosphate-dependent) OS=Brachypodium distachyon	-2.11
A0A3B6HT18	Phosphomannomutase OS=Triticum aestivum	-1.31
A0A5J9WER5	Photosystem I assembly protein Ycf4 (Fragment) OS=Eragrostis curvula	-1.45
A0A2U9DRJ5	Photosystem I P700 chlorophyll a apoprotein A1 OS=Campeiostachys nutans	-1.38
A0A4P8F6B8	Photosystem I P700 chlorophyll a apoprotein A2 OS=Triodia mallota	-0.73
A0A5P8FSR9	Photosystem II protein D1 OS=Bambusa variostriata	-0.94
A0A0E0QCR5	Plant heme peroxidase family profile domain-containing protein OS=Oryza rufipogon	-2.42
O64411	Polyamine oxidase 1 OS=Zea mays	-2.06
Q2QP54	Polyprotein of EF-Ts, chloroplastic OS=Oryza sativa subsp. japonica	-1.62
A0A0E0NUQ9	Potassium transporter OS=Oryza rufipogon	-1.01
Q2RAK2	Pyruvate kinase 1, cytosolic OS=Oryza sativa subsp. japonica	1.01
A0A0E0HXE6	Pyruvate kinase OS=Oryza nivara	-1.6
A0A3B5Y1F9	Pyruvate, phosphate dikinase OS=Triticum aestivum	2.17

A0A2S3ID39	Receptor-like serine/threonine-protein kinase OS=Panicum hallii	-1.92
A0A2T7CH14	Ribulose-phosphate 3-epimerase OS=Panicum hallii var. hallii	-1.18
A0A0E0D0N3	RING-type E3 ubiquitin transferase OS=Oryza meridionalis	-0.99
A0A0E0F9Y5	RNA cytidine acetyltransferase OS=Oryza meridionalis	0.65
A0A077RWS5	S-adenosylmethionine synthase OS=Triticum aestivum	-1.03
P46285	Sedoheptulose-1,7-bisphosphatase, chloroplastic OS=Triticum aestivum	-2
A0A0C4BJE5	Serine hydroxymethyltransferase OS=Triticum aestivum	-0.38
P49027	Small ribosomal subunit protein RACK1z OS=Oryza sativa subsp. japonica	-1.47
Q6YXW6	Sucrose-phosphatase 2 OS=Oryza sativa subsp. japonica	2.31
P09233	Superoxide dismutase [Mn] 3.1, mitochondrial OS=Zea mays	0.64
A0A3B6RMJ1	Thiamine thiazole synthase, chloroplastic OS=Triticum aestivum	-4
A0A0Q3NCX3	Thioredoxin reductase OS=Brachypodium distachyon	1.28
A0A3L6R8U7	threonine synthase OS=Panicum miliaceum	0.44
Q7SIC9	Transketolase, chloroplastic OS=Zea mays	-1.66
Q9ZRB0	Tubulin beta-3 chain OS=Triticum aestivum	3.26
A0A0U2GJM5	UDP-arabinopyranose mutase OS=Hordeum vulgare	1.58
Q6Z7B0	Heat shock 70 kDa protein BIP1 OS=Oryza sativa subsp. japonica	1.19
Q6Z058	Heat shock 70 kDa protein BIP5 OS=Oryza sativa subsp. japonica	2.23
A0A0E0PRJ8	very-long-chain 3-oxoacyl-CoA synthase	1.75
A0A1D6FIR4	V-type proton ATPase proteolipid subunit (Fragment) OS=Zea mays	-2.54
Q8L5C6	Xylanase inhibitor protein 1 OS=Triticum aestivum	1.59

Table S8 List of differentially expressed proteins with their accession ID, description and log2 fold change (logFC) when comparing heat stress condition against control condition for S11 729-10 at 14 d

Accession	Description	logFC
P30792	2,3-bisphosphoglycerate-independent phosphoglycerate mutase OS=Zea mays	-1.3
W5EP13	2-carboxy-D-arabinitol-1-phosphatase OS=Triticum aestivum	1.71
A0A3L6E9M0	4-coumarate--CoA ligase OS=Zea mays	-0.64
A0A2S3IJL2	5-methyltetrahydropteroyltriglutamate--homocysteine S-methyltransferase OS=Panicum hallii	-2.86
A0A3B6TBU2	6-phosphogluconate dehydrogenase, decarboxylating OS=Triticum aestivum	-0.86
A0A2T7DTW1	AAA+ ATPase domain-containing protein OS=Panicum hallii var. hallii	-1.4
A0A3B6NJT0	Acetyltransferase component of pyruvate dehydrogenase complex OS=Triticum aestivum	0.84
A0A0Q3F6L1	Aconitate hydratase OS=Brachypodium distachyon	-0.58
A0A194YR04	Adenosine kinase OS=Sorghum bicolor	-0.84
P32112	Adenosylhomocysteinase OS=Triticum aestivum	-2.45
A0A3B6JPU8	Adenylosuccinate synthetase, chloroplastic OS=Triticum aestivum	-0.99
A0A1D6HR58	alanine transaminase OS=Zea mays	-1.63
Q0DWH1	Alcohol dehydrogenase class-3 OS=Oryza sativa subsp. japonica	0.9
A0A1B6QHW4	Alpha-1,4 glucan phosphorylase OS=Sorghum bicolor	-1.56
Q9FXT4	Alpha-galactosidase OS=Oryza sativa subsp. japonica	-2.58
C6KEM4	Aminoaldehyde dehydrogenase 2 OS=Zea mays	-1.77
A0A453NS22	Aminopeptidase OS=Aegilops tauschii subsp. strangulata	-0.58
B8AU84	Arginase 1, mitochondrial OS=Oryza sativa subsp. indica	2.02
P37833	Aspartate aminotransferase, cytoplasmic OS=Oryza sativa subsp. japonica	-1.99
A0A1B6PGM0	aspartate carbamoyltransferase OS=Sorghum bicolor	0.7
A0A3B5ZZW5	assimilatory sulfite reductase (ferredoxin) OS=Triticum aestivum	-2.09
A0A2L0VAS4	ATP synthase subunit alpha, chloroplastic OS=Lamarckia aurea	-1.04
A0A2L0VAT4	ATP synthase subunit beta, chloroplastic OS=Lamarckia aurea	-1.48
P0C1M0	ATP synthase subunit gamma, chloroplastic OS=Zea mays	-1.11
A0A0H3V9P6	ATP-dependent Clp protease proteolytic subunit OS=Oryza glumipatula	0.65
A0A317YBF8	ATP-dependent DNA helicase OS=Zea mays	2.42
P16098	Beta-amylase OS=Hordeum vulgare	-3.44
Q84LK3	Betaine aldehyde dehydrogenase 2 OS=Oryza sativa subsp. japonica	-1.24
P04464	Calmodulin OS=Triticum aestivum	-1.03
A0A3B6KQP3	Catalase OS=Triticum aestivum	1.08

A0A1W0VZF1	CBM20 domain-containing protein OS=Sorghum bicolor	2.94
P29185	Chaperonin CPN60-1, mitochondrial OS=Zea mays	2.12
A0A0E0GTN2	Cysteine synthase OS=Oryza nivara	-1.49
A0A3G1AT48	Cytochrome b6 OS=Bromus vulgaris	-0.46
Q7X9A6	Cytochrome b6-f complex iron-sulfur subunit, chloroplastic OS=Triticum aestivum	-1.09
A0A0E0E626	Delta-aminolevulinic acid dehydratase OS=Oryza meridionalis	0.84
A0A0Q3FJF1	dihydroxy-acid dehydratase OS=Brachypodium distachyon	-0.75
A0A0E0JJD7	Endopeptidase Clp OS=Oryza punctata	-1.98
P36183	Endoplasmin homolog OS=Hordeum vulgare	1.4
A4KAG8	Ent-isokaur-15-ene synthase OS=Oryza sativa subsp. japonica	-1.27
Q6Z2Z4	Eukaryotic initiation factor 4A-3 OS=Oryza sativa subsp. japonica	-0.56
Q69RJ0	Ferredoxin-dependent glutamate synthase, chloroplastic	-1.29
P41344	Ferredoxin--NADP reductase, leaf isozyme 1, chloroplastic	-2.15
Q6ZFJ3	Ferredoxin--NADP reductase, leaf isozyme 2, chloroplastic	-2.65
A0A0E0N8J2	ferredoxin--NADP(+) reductase OS=Oryza rufipogon	-2.06
Q42997	Ferredoxin--nitrite reductase, chloroplastic OS=Oryza sativa subsp. japonica	-2.24
A0A1D5YQ12	Ferritin OS=Triticum aestivum	1.37
Q84N28	Flavone O-methyltransferase 1 OS=Triticum aestivum	-2.5
Q0JGZ6	Fructokinase-1 OS=Oryza sativa subsp. japonica	-0.84
Q0J8G4	Fructokinase-2 OS=Oryza sativa subsp. japonica	-2.33
A0A2K2DTT5	fructose-bisphosphatase OS=Brachypodium distachyon	-0.6
P17784	Fructose-bisphosphate aldolase 1, cytoplasmic OS=Oryza sativa subsp. japonica	-1.03
Q40677	Fructose-bisphosphate aldolase, chloroplastic OS=Oryza sativa subsp. japonica	0.7
Q7XN11	Gamma-aminobutyrate transaminase 1, mitochondrial OS=Oryza sativa subsp. japonica	-1.06
A3C4S4	GDP-mannose 3,5-epimerase 1 OS=Oryza sativa subsp. japonica	-1.41
Q6ZBZ2	Germin-like protein 8-14 OS=Oryza sativa subsp. japonica	-1.85
Q6AVT2	Glucose-1-phosphate adenylyltransferase large subunit 1, chloroplastic/amyloplastic OS=Oryza sativa subsp. japonica	-1.61
P55239	Glucose-1-phosphate adenylyltransferase large subunit 2 (Fragment) OS=Hordeum vulgare	-1.45
A0A3B6KSU0	Glucose-1-phosphate adenylyltransferase OS=Triticum aestivum	-1.29
A0A1D6I644	Glucose-6-phosphate isomerase OS=Zea mays	-0.96
Q33E23	Glutamate dehydrogenase 2, mitochondrial OS=Oryza sativa subsp. japonica	-1.75
I1HQF1	glutamate synthase (NADH) OS=Brachypodium distachyon	-2.91
P38562	Glutamine synthetase root isozyme 4 OS=Zea mays	-1.9
A0A1D6HW14	Glutaredoxin-dependent peroxiredoxin OS=Zea mays	-0.72
A0A3B6JM67	Glutathione reductase OS=Triticum aestivum	-0.94

P08735	Glyceraldehyde-3-phosphate dehydrogenase 1, cytosolic OS=Zea mays	-0.34
Q7FAH2	Glyceraldehyde-3-phosphate dehydrogenase 2, cytosolic OS=Oryza sativa subsp. japonica	-0.91
Q43247	Glyceraldehyde-3-phosphate dehydrogenase 3, cytosolic OS=Zea mays	-1.13
P09315	Glyceraldehyde-3-phosphate dehydrogenase A, chloroplastic OS=Zea mays	-0.5
Q10CE4	Glycolate oxidase 1 OS=Oryza sativa subsp. japonica	-1.4
B8B7C5	Glycolate oxidase 5 OS=Oryza sativa subsp. indica	-1.2
A0A0P0VLJ4	GrpE protein homolog OS=Oryza sativa subsp. japonica	0.98
A0A3L6RHP0	GTP 3',8-cyclase OS=Panicum miliaceum	-0.95
C5WVT9	Guanine nucleotide-binding protein alpha subunit OS=Sorghum bicolor	-1.5
Q0J4P2	Heat shock protein 81-1 OS=Oryza sativa subsp. japonica	0.93
P02276	Histone H2A.2.1 OS=Triticum aestivum	-2.02
P02277	Histone H2A.2.2 OS=Triticum aestivum	-1.98
A2WKT4	Histone H2B.5 OS=Oryza sativa subsp. indica	-2.28
P68428	Histone H3.2 OS=Triticum aestivum	-2.82
Q0JCT1	Histone H3.3 OS=Oryza sativa subsp. japonica	-2.66
P62787	Histone H4 OS=Zea mays	-2.62
A0A3B6LUD2	Inosine-5'-monophosphate dehydrogenase OS=Triticum aestivum	-0.9
O64437	Inositol-3-phosphate synthase 1 OS=Oryza sativa subsp. japonica	-1.98
A0A3B6GTN3	Isocitrate dehydrogenase [NADP] OS=Triticum aestivum	-0.43
A0A3B5Z5S1	Ketol-acid reductoisomerase OS=Triticum aestivum	-0.8
P17788	Large ribosomal subunit protein uL2cz/uL2cy OS=Zea mays	-1.1
Q9FE01	L-ascorbate peroxidase 2, cytosolic OS=Oryza sativa subsp. japonica	-0.48
Q6K669	Leucine aminopeptidase 2, chloroplastic OS=Oryza sativa subsp. japonica	-0.69
P24067	Luminal-binding protein 2 OS=Zea mays	1.18
Q9SE94	Methylenetetrahydrofolate reductase (NADH) 1 OS=Zea mays	-0.95
A0A0E0EWM9	Mevalonate kinase OS=Oryza meridionalis	-1.25
Q652L6	Monodehydroascorbate reductase 3, cytosolic OS=Oryza sativa subsp. japonica	-0.89
A0A0E0D501	Multifunctional fusion protein OS=Oryza meridionalis	0.74
Q8LK61	NADP-dependent glyceraldehyde-3-phosphate dehydrogenase OS=Triticum aestivum	-1.01
P43279	NADP-dependent malic enzyme, chloroplastic OS=Oryza sativa subsp. japonica	-3.19
B4FK49	Nucleoside diphosphate kinase 1 OS=Zea mays	-1.81
A0A0Q3RM53	ornithine aminotransferase OS=Brachypodium distachyon	2.02
A0A3B6LEJ3	Pectinesterase OS=Triticum aestivum	-3.49

Q336R9	Peptide methionine sulfoxide reductase A4, chloroplastic OS=Oryza sativa subsp. japonica	1.17
P27337	Peroxidase 1 OS=Hordeum vulgare	-1.11
A0A453APJ3	Peroxidase OS=Aegilops tauschii subsp. strangulata	-1.23
Q9FR35	Peroxiredoxin-2C OS=Oryza sativa subsp. japonica	-1.71
A0A0Q3JDL9	phosphoglucomutase (alpha-D-glucose-1,6-bisphosphate-dependent) OS=Brachypodium distachyon	-3.16
A0A3B6JK87	phosphoglycerate mutase (2,3-diphosphoglycerate-independent) OS=Triticum aestivum	-1.46
Q43007	Phospholipase D alpha 1 OS=Oryza sativa subsp. japonica	-1.32
A0A3B6HT18	Phosphomannomutase OS=Triticum aestivum	-2.86
A0A2U9DRJ5	Photosystem I P700 chlorophyll a apoprotein A1 OS=Campeiostachys nutans	-0.82
Q0J8R9	Photosystem II 22 kDa protein 2, chloroplastic OS=Oryza sativa subsp. japonica	-0.88
A0A218LWN1	Photosystem II CP43 reaction center protein OS=Triticum monococcum	-0.69
A0A0S1S3S5	Photosystem II D2 protein OS=Stipa lipskyi	-0.84
A0A5P8FSR9	Photosystem II protein D1 OS=Bambusa variostriata	-0.93
A0A0E0QCR5	Plant heme peroxidase family profile domain-containing protein OS=Oryza rufipogon	-1.72
Q2QP54	Polyprotein of EF-Ts, chloroplastic OS=Oryza sativa subsp. japonica	-1.55
Q6H6D2	Porphobilinogen deaminase, chloroplastic OS=Oryza sativa subsp. japonica	-1.69
A0A0E0NUQ9	Potassium transporter OS=Oryza rufipogon	-2.29
P80607	Probable UDP-arabinopyranose mutase 1 OS=Zea mays	-1.87
O22655	Profilin-4 OS=Zea mays	-1.53
P17070	Proliferating cell nuclear antigen OS=Oryza sativa subsp. japonica	-0.88
A0A3B6RCJ8	Proteasome subunit beta OS=Triticum aestivum	-0.97
A0A0E0NKR5	Pyruvate dehydrogenase E1 component subunit alpha OS=Oryza rufipogon	-0.83
A0A0E0HXE6	Pyruvate kinase OS=Oryza nivara	-1.51
A0A2S3ID39	Receptor-like serine/threonine-protein kinase OS=Panicum hallii	-1.48
A0A3L6DQ42	Ribokinase OS=Zea mays	-1.95
A0A8A6P3J7	Ribulose bisphosphate carboxylase large chain OS=Sphenopholis intermedia	0.62
A0A0E0MLI8	Ribulose bisphosphate carboxylase small subunit, chloroplastic OS=Oryza punctata	0.65
A0A2T7CH14	Ribulose-phosphate 3-epimerase OS=Panicum hallii var. hallii	-1.21
A0A0E0D0N3	RING-type E3 ubiquitin transferase OS=Oryza meridionalis	-0.98
A0A077RWS5	S-adenosylmethionine synthase OS=Triticum aestivum	-2.22
P46285	Sedoheptulose-1,7-bisphosphatase, chloroplastic OS=Triticum aestivum	-2.27

A0A0C4BJE5	Serine hydroxymethyltransferase OS=Triticum aestivum	-1.13
P49027	Small ribosomal subunit protein RACK1z OS=Oryza sativa subsp. japonica	-1.86
A0A1D6Q567	Succinate--CoA ligase [ADP-forming] subunit beta, mitochondrial OS=Zea mays	-0.44
P30298	Sucrose synthase 2 OS=Oryza sativa subsp. japonica	-0.69
A0A3B6RMJ1	Thiamine thiazole synthase, chloroplastic OS=Triticum aestivum	-3.86
A0A0Q3NCX3	Thioredoxin reductase OS=Brachypodium distachyon	0.4
Q7SIC9	Transketolase, chloroplastic OS=Zea mays	-1.99
P46225	Triosephosphate isomerase, chloroplastic OS=Secale cereale	-1.38
Q9ZRB0	Tubulin beta-3 chain OS=Triticum aestivum	0.53
A0A0Q3KYB0	Ubiquitin-like domain-containing protein OS=Brachypodium distachyon	-0.62
Q8H8T0	UDP-arabinopyranose mutase 1 OS=Oryza sativa subsp. japonica	-1.91
J3LV62	UMP-CMP kinase OS=Oryza brachyantha	-2.35
Q6Z7B0	Heat shock 70 kDa protein BIP1 OS=Oryza sativa subsp. japonica	0.72
Q6Z058	Heat shock 70 kDa protein BIP5 OS=Oryza sativa subsp. japonica	1.36
A0A0E0PRJ8	very-long-chain 3-oxoacyl-CoA synthase OS=Oryza rufipogon	0.6
P49087	V-type proton ATPase catalytic subunit A (Fragment) OS=Zea mays	-0.96
A0A1D6FIR4	V-type proton ATPase proteolipid subunit (Fragment) OS=Zea mays	-1.31

Table S9 List of differentially expressed proteins with their accession ID, description and log2 fold change (logFC) when comparing heat stress condition against control condition for S11 729-10 at 28 d

Accession	Description	logFC
P30792	2,3-bisphosphoglycerate-independent phosphoglycerate mutase OS=Zea mays	-1
W5EP13	2-carboxy-D-arabinitol-1-phosphatase OS=Triticum aestivum	2.61
Q337M4	2-oxoadipate dioxygenase/decarboxylase, chloroplastic/amyloplastic OS=Oryza sativa subsp. japonica	2.27
Q6AVG6	4-hydroxy-3-methylbut-2-enyl diphosphate reductase, chloroplastic OS=Oryza sativa subsp. japonica	0.66
A0A2S3IJL2	5-methyltetrahydropteroyltriglutamate--homocysteine S-methyltransferase OS=Panicum hallii	-2.17
A0A3B6TBU2	6-phosphogluconate dehydrogenase, decarboxylating OS=Triticum aestivum	-0.59
A0A3B6NJT0	Acetyltransferase component of pyruvate dehydrogenase complex OS=Triticum aestivum	1.82
P32112	Adenosylhomocysteinase OS=Triticum aestivum	-2.06
A0A3B6JPU8	Adenylosuccinate synthetase, chloroplastic OS=Triticum aestivum	-0.82
A0A1D6HR58	alanine transaminase OS=Zea mays	-1.76
A0A1B6QHW4	Alpha-1,4 glucan phosphorylase OS=Sorghum bicolor	-1.63
Q9FXT4	Alpha-galactosidase OS=Oryza sativa subsp. japonica	-1.87
B8AU84	Arginase 1, mitochondrial OS=Oryza sativa subsp. indica	2.47
P37833	Aspartate aminotransferase, cytoplasmic OS=Oryza sativa subsp. japonica	-1.81
A0A1B6PGM0	aspartate carbamoyltransferase OS=Sorghum bicolor	1.25
A0A0E0D753	aspartate-semialdehyde dehydrogenase OS=Oryza meridionalis	0.68
A0A3B5ZZW5	assimilatory sulfite reductase (ferredoxin) OS=Triticum aestivum	-2.2
A0A2L0VAT4	ATP synthase subunit beta, chloroplastic OS=Lamarckia aurea	-1.43
P0C1M0	ATP synthase subunit gamma, chloroplastic OS=Zea mays	-1.19
A0A317YBF8	ATP-dependent DNA helicase OS=Zea mays	3.78
P16098	Beta-amylase OS=Hordeum vulgare	-3.45
A0A3B5Y061	Branched-chain-amino-acid aminotransferase OS=Triticum aestivum	0.65
B9EXM2	Carbamoyl-phosphate synthase large chain, chloroplastic OS=Oryza sativa subsp. japonica	0.97
A0A3B6KQP3	Catalase OS=Triticum aestivum	1.98
A0A1W0VZF1	CBM20 domain-containing protein OS=Sorghum bicolor	3.88
P29185	Chaperonin CPN60-1, mitochondrial OS=Zea mays	3.25
A2XJ35	Chlorophyll a-b binding protein, chloroplastic OS=Oryza sativa subsp. indica	0.51
A0A0E0GTN2	Cysteine synthase OS=Oryza nivara	-0.61
Q0JM17	DEAD-box ATP-dependent RNA helicase 56 OS=Oryza sativa subsp. japonica	0.87

A0A0E0JJD7	Endopeptidase Clp OS=Oryza punctata	-1.46
P36183	Endoplasmin homolog OS=Hordeum vulgare	1.39
Q69RJ0	Ferredoxin-dependent glutamate synthase, chloroplastic OS=Oryza sativa subsp. japonica	-1.18
P41344	Ferredoxin--NADP reductase, leaf isozyme 1, chloroplastic OS=Oryza sativa subsp. japonica	-1.96
Q6ZFJ3	Ferredoxin--NADP reductase, leaf isozyme 2, chloroplastic OS=Oryza sativa subsp. japonica	-2.47
A0A0E0N8J2	ferredoxin--NADP(+) reductase OS=Oryza rufipogon	-1.86
Q42997	Ferredoxin--nitrite reductase, chloroplastic OS=Oryza sativa subsp. japonica	-1.56
A0A1D5YQ12	Ferritin OS=Triticum aestivum	1.03
Q84N28	Flavone O-methyltransferase 1 OS=Triticum aestivum	-2.29
A0A4V6DBK4	Formate dehydrogenase, mitochondrial OS=Setaria viridis	1.65
Q0JGZ6	Fructokinase-1 OS=Oryza sativa subsp. japonica	-0.42
A0A2K2DTT5	fructose-bisphosphatase OS=Brachypodium distachyon	-0.47
Q40677	Fructose-bisphosphate aldolase, chloroplastic OS=Oryza sativa subsp. japonica	1.35
Q6AVT2	Glucose-1-phosphate adenylyltransferase large subunit 1, chloroplastic/amyloplastic OS=Oryza sativa subsp. japonica	-1.08
A0A3B6KSU0	Glucose-1-phosphate adenylyltransferase OS=Triticum aestivum	-1.06
A0A1D6I644	Glucose-6-phosphate isomerase OS=Zea mays	-0.99
Q33E23	Glutamate dehydrogenase 2, mitochondrial OS=Oryza sativa subsp. japonica	-1.98
I1HQF1	glutamate synthase (NADH) OS=Brachypodium distachyon	-3.15
P38562	Glutamine synthetase root isozyme 4 OS=Zea mays	-2.04
A0A3B6JM67	Glutathione reductase OS=Triticum aestivum	-0.59
Q10CE4	Glycolate oxidase 1 OS=Oryza sativa subsp. japonica	-0.97
B8B7C5	Glycolate oxidase 5 OS=Oryza sativa subsp. indica	-0.83
A0A0P0VLJ4	GrpE protein homolog OS=Oryza sativa subsp. japonica	1.46
C5WVT9	Guanine nucleotide-binding protein alpha subunit OS=Sorghum bicolor	-1.26
Q0J4P2	Heat shock protein 81-1 OS=Oryza sativa subsp. japonica	1.66
P02276	Histone H2A.2.1 OS=Triticum aestivum	-1.78
A2WKT4	Histone H2B.5 OS=Oryza sativa subsp. indica	-1.67
P68428	Histone H3.2 OS=Triticum aestivum	-2.51
Q0JCT1	Histone H3.3 OS=Oryza sativa subsp. japonica	-2.06
P62787	Histone H4 OS=Zea mays	-2.26
F8RP11	Hsp70-Hsp90 organizing protein OS=Triticum aestivum	1.46
P17788	Large ribosomal subunit protein uL2cz/uL2cy OS=Zea mays	-1.17
A0A3B6PVU5	L-ascorbate peroxidase OS=Triticum aestivum	1.12
P24067	Luminal-binding protein 2 OS=Zea mays	1.71
Q652L6	Monodehydroascorbate reductase 3, cytosolic OS=Oryza sativa subsp. japonica	-0.66

A0A0E0D501	Multifunctional fusion protein OS=Oryza meridionalis	1.04
P43279	NADP-dependent malic enzyme, chloroplastic OS=Oryza sativa subsp. japonica	-2.52
A0A0Q3RM53	ornithine aminotransferase OS=Brachypodium distachyon	2.19
A0A3B6LEJ3	Pectinesterase OS=Triticum aestivum	-2.9
Q336R9	Peptide methionine sulfoxide reductase A4, chloroplastic OS=Oryza sativa subsp. japonica	1.18
A0A3B6HT18	Phosphomannomutase OS=Triticum aestivum	-1.73
Q943K1	Photosystem II 22 kDa protein 1, chloroplastic OS=Oryza sativa subsp. japonica	0.74
A0A218LWN1	Photosystem II CP43 reaction center protein OS=Triticum monococcum	-0.63
A0A0S1S3S5	Photosystem II D2 protein OS=Stipa lipskyi	-0.71
A0A5P8FSR9	Photosystem II protein D1 OS=Bambusa variostriata	-0.79
O64411	Polyamine oxidase 1 OS=Zea mays	-1.29
Q67UK9	Probable glutathione S-transferase DHAR2, chloroplastic OS=Oryza sativa subsp. japonica	1.16
Q69SV0	Probable L-ascorbate peroxidase 8, chloroplastic OS=Oryza sativa subsp. japonica	0.96
P80607	Probable UDP-arabinopyranose mutase 1 OS=Zea mays	-1.35
O22655	Profilin-4 OS=Zea mays	-1.4
A0A3B6RCJ8	Proteasome subunit beta OS=Triticum aestivum	-0.77
A0A0E0NKR5	Pyruvate dehydrogenase E1 component subunit alpha OS=Oryza rufipogon	-0.77
A0A0E0HXE6	Pyruvate kinase OS=Oryza nivara	-1.45
A0A2S3ID39	Receptor-like serine/threonine-protein kinase OS=Panicum hallii	-1.29
A0A0E0MLI8	Ribulose bisphosphate carboxylase small subunit, chloroplastic OS=Oryza punctata	0.34
A0A0E0F9Y5	RNA cytidine acetyltransferase OS=Oryza meridionalis OX=40149 PE=3 SV=1	1.22
A0A077RWS5	S-adenosylmethionine synthase OS=Triticum aestivum	-1.72
P46285	Sedoheptulose-1,7-bisphosphatase, chloroplastic OS=Triticum aestivum	-2.09
P49027	Small ribosomal subunit protein RACK1z OS=Oryza sativa subsp. japonica	-1.79
B9F3B6	Succinate-semialdehyde dehydrogenase, mitochondrial OS=Oryza sativa subsp. japonica	1.03
A0A0D3H388	Superoxide dismutase [Cu-Zn] OS=Oryza barthii	-1.03
P09233	Superoxide dismutase [Mn] 3.1, mitochondrial OS=Zea mays	1.32
A0A3B6RMJ1	Thiamine thiazole synthase, chloroplastic OS=Triticum aestivum	-3.32
Q7SIC9	Transketolase, chloroplastic OS=Zea mays	-1.83
P46225	Triosephosphate isomerase, chloroplastic OS=Secale cereale	-1.13
Q9ZRB0	Tubulin beta-3 chain OS=Triticum aestivum	2.06
Q8H8T0	UDP-arabinopyranose mutase 1 OS=Oryza sativa subsp. japonica	-1.03
J3LV62	UMP-CMP kinase OS=Oryza brachyantha	-1.59

Q6Z058	Heat shock 70 kDa protein BIP5 OS=Oryza sativa subsp. japonica	2.43
A0A0E0PRJ8	very-long-chain 3-oxoacyl-CoA synthase OS=Oryza rufipogon	1.07
P49087	V-type proton ATPase catalytic subunit A (Fragment) OS=Zea mays	-0.6
A0A1D6FIR4	V-type proton ATPase proteolipid subunit (Fragment) OS=Zea mays	-1.6

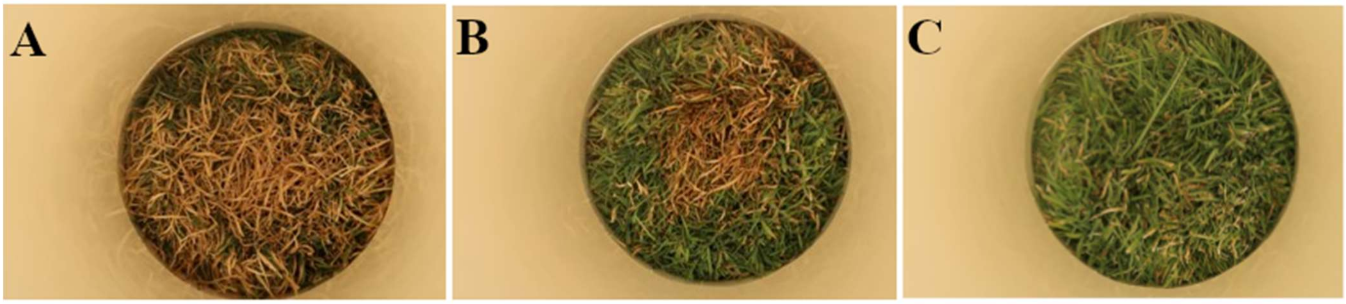

Figure S1. Representative pots of three creeping bentgrass lines including Crenshaw (A), S11 675-02 (B) and S11 729-10 (C) at 28 d of heat stress (38/33°C day/night).

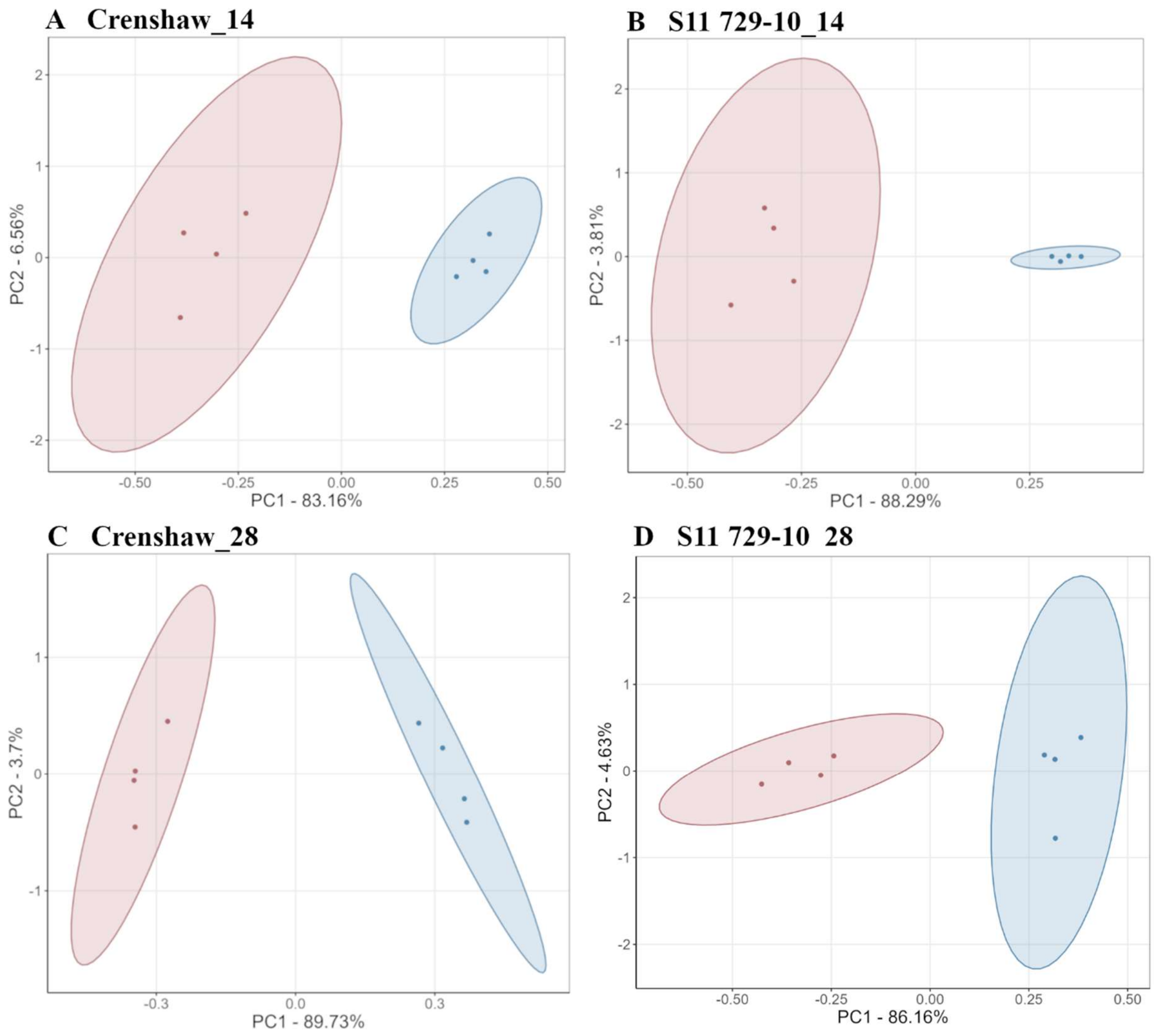

Figure S2. Principal component analysis for differentially expressed proteins of two creeping bentgrass lines (Crenshaw and S11 729-10) at 14 d (A, B) and 28 d (C, D). The red circle represented the control group while the blue circle represented the heat-stressed group within each plot.

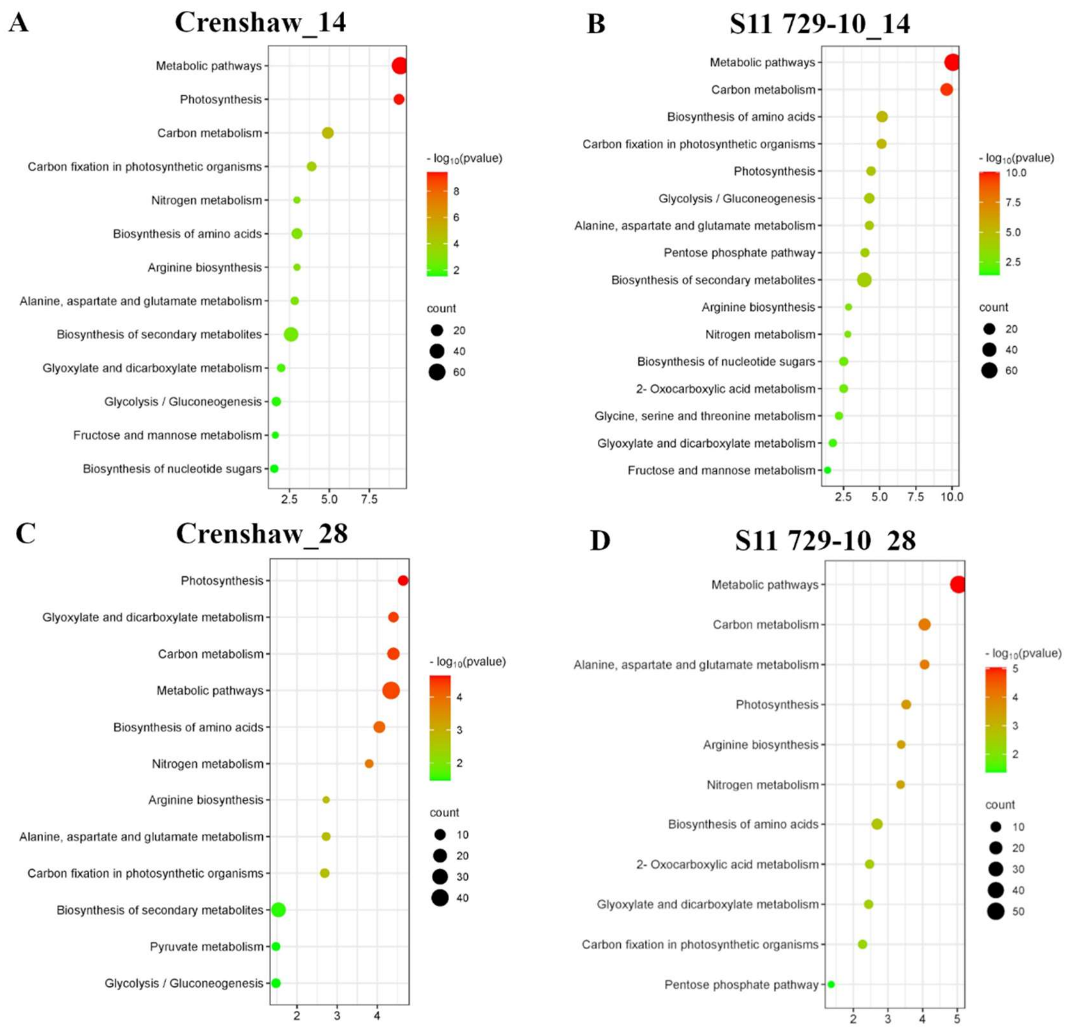

Figure S3. Kyoto Encyclopedia of Genes and Genomes (KEGG) analysis for differentially expressed proteins of two creeping bentgrass lines (Crenshaw and S11 729-10) at 14 d (A, B) and 28 d (C, D) responding to heat stress. The X axis represented the values of $-\log_{10}$(pvalue).
